# Hypoxia drives the assembly of the multienzyme purinosome complex

**DOI:** 10.1074/jbc.RA119.012175

**Published:** 2020-05-21

**Authors:** Cyrielle Doigneaux, Anthony M. Pedley, Ishna N. Mistry, Monika Papayova, Stephen J. Benkovic, Ali Tavassoli

**Affiliations:** 1 School of Chemistry, University of Southampton, Southampton, United Kingdom; 2 Department of Chemistry, The Pennsylvania State University, University Park, Pennsylvania, USA

**Keywords:** purinosome, hypoxia, hypoxia-inducible factor 1, HIF-1, metabolon, *de novo* purine biosynthesis, 5-aminoimidazole-4-carboxamide ribonucleotide formyltransferase/IMP cyclohydrolase (ATIC), cellular metabolism, purine, hypoxia-inducible factor (HIF), cell metabolism, metabolism

## Abstract

The purinosome is a dynamic metabolic complex composed of enzymes responsible for *de novo* purine biosynthesis, whose formation has been associated with elevated purine demand. However, the physiological conditions that govern purinosome formation in cells remain unknown. Here, we report that purinosome formation is up-regulated in cells in response to a low-oxygen microenvironment (hypoxia). We demonstrate that increased purinosome assembly in hypoxic human cells requires the activation of hypoxia inducible factor 1 (HIF-1) and not HIF-2. Hypoxia-driven purinosome assembly was inhibited in cells lacking 5-aminoimidazole-4-carboxamide ribonucleotide formyltransferase/IMP cyclohydrolase (ATIC), a single enzyme in *de novo* purine biosynthesis, and in cells treated with a small molecule inhibitor of ATIC homodimerization. However, despite the increase in purinosome assembly in hypoxia, we observed no associated increase in *de novo* purine biosynthesis in cells. Our results indicate that this was likely due to a reduction in mitochondrial one-carbon metabolism, resulting in reduced mitochondrion-derived one-carbon units needed for *de novo* purine biosynthesis. The findings of our study further clarify and deepen our understanding of purinosome formation by revealing that this process does not solely depend on cellular purine demand.

Purines are more than just the building blocks for DNA and RNA; they are key metabolites that are critical for cellular function. Purines constitute the cellular energy unit ATP, the key signaling molecule GTP, and the substrates and cofactors for a variety of cellular pathways. There are two paths for purine production in cells: recycling of existing bases via a salvage pathway and *de novo* synthesis of the purine precursor inosine monophosphate (IMP) from phosphoribosyl pyrophosphate (PRPP) by six enzymes in 10 steps. Purine salvage is the predominant path for purine production in nonmalignant human cells as it is more resource efficient (1). It has been hypothesized that during periods of rapid cell growth, the *de novo* purine biosynthesis pathway is up-regulated, but little is known about other factors that affect the balance between these two pathways (1).

As with other multienzyme pathways, it is difficult to explain the intracellular kinetics of *de novo* purine biosynthesis and the chemical stability of several intermediates if the six enzymes of this pathway were randomly dispersed within the cytosol. The association of these enzymes in a functional multienzyme complex or “metabolon” has therefore been a longstanding hypothesis. Using a combination of microscopy-based techniques, the six *de novo* purine biosynthetic enzymes were shown to assemble into a dynamic complex in cells named the purinosome, in response to purine depletion from the cell culture medium (2–4). Purinosome formation has been correlated with an increase in *de novo* purine biosynthesis, suggesting the functional relevance of this process (5). Several studies have demonstrated that purinosome assembly may be disrupted in cells with microtubule polymerization inhibitors and with functional mutations in ATIC and adenylosuccinate lyase (ADSL) (6–8). However, little is known about physiological conditions that trigger purinosome formation.

One physiological factor that significantly alters cell metabolism is hypoxia. Cellular adaptation to hypoxia is orchestrated by HIF-1, a heterodimeric transcription factor that is composed of an oxygen-regulated α-subunit and a constitutively expressed β-subunit (9). HIF-1α has a *t*_1/2_ of less than 5 min in normoxia (9). Prolyl hydroxylase enzymes catalyze the hydroxylation of two proline residues (Pro-402 and Pro-564) using oxygen as a substrate, marking HIF-1α for ubiquitination and degradation (10, 11). This process does not occur in the absence of molecular oxygen, the substrate for the enzyme, leading to the rapid build-up of HIF-1α and its translocation to the nucleus, where its binds to HIF-1β to form the active HIF-1 transcription factor. Thus, the HIF-1 response to hypoxia is near instantaneous, with HIF-1α both sensing and responding to reduced cellular oxygen levels. HIF-1 enables cellular adaptation to hypoxia by reprogramming the expression of hundreds of genes, including those associated with several metabolic pathways (12–14). Glycolysis and the pentose phosphate pathway (PPP) are known to be up-regulated in hypoxia, whereas the tricarboxylic acid cycle is down-regulated (15–18). Prior studies have also linked colocalization of glycolytic enzymes in response to hypoxia (19, 20). Although the effect of HIF-1 on cellular energy production is well-characterized, less is known about the effect of hypoxia on other metabolic pathways (16–18). Up-regulation of glycolysis and PPP in hypoxia serves to increase metabolites that feed into various pathways to synthesize key molecules required for cell proliferation (21). As purinosome formation has been correlated to increased *de novo* purine biosynthesis, and considering the significant metabolic reprogramming that occurs in hypoxic cells, we hypothesized that a hypoxic environment would lead to increased purinosome formation in cells.

## Results

### Hypoxia drives purinosome assembly

**Figure 1.**
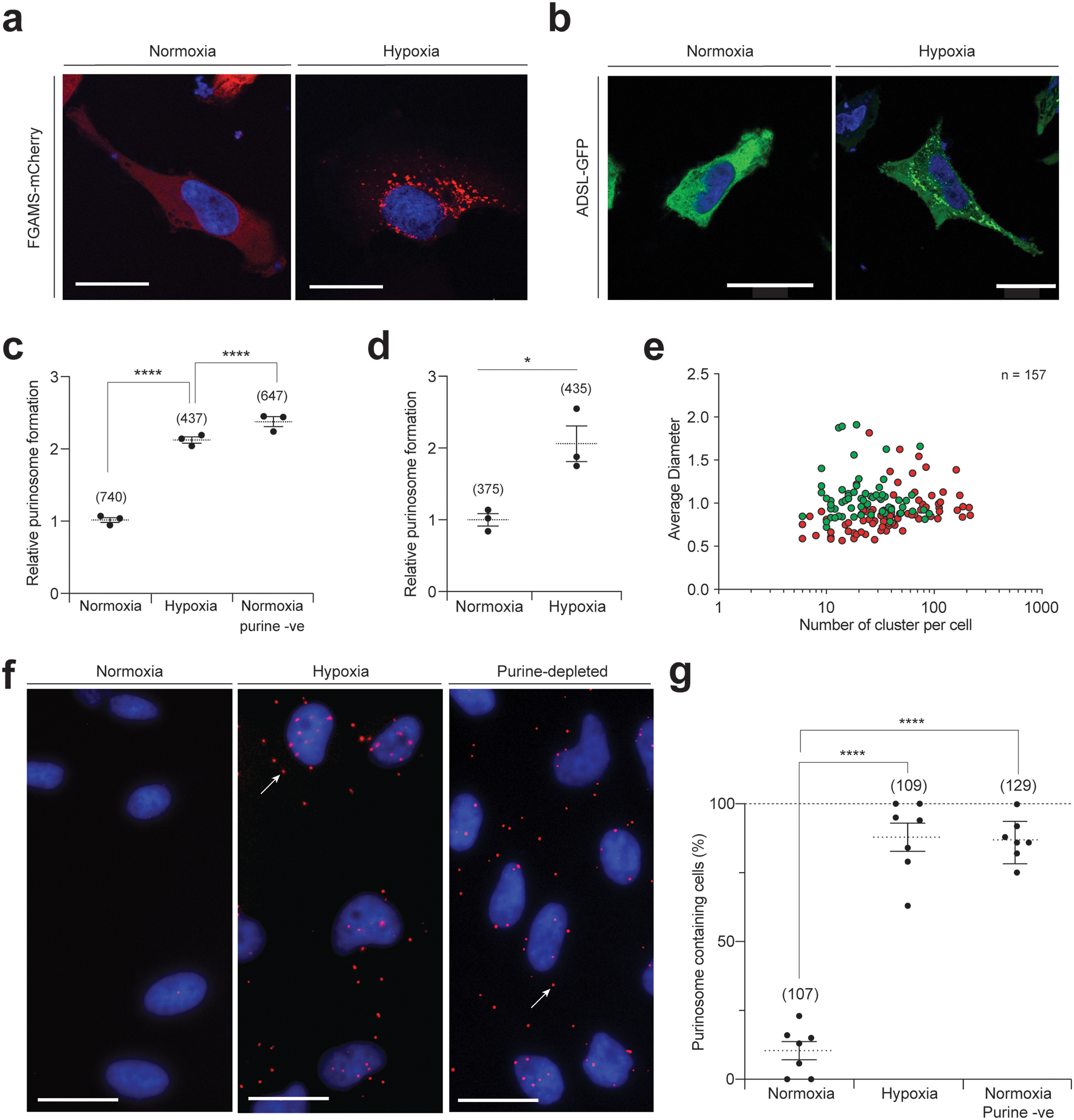
**The formation of the purinosome complex in hypoxic HeLa cells.** *a,* visualizing purinosome formation in hypoxic cells using mCherry-tagged FGAMS. Fluorescent clusters can be observed in hypoxic cells. The DAPI-stained nuclei are shown in *blue*. *Scale bar* = 25 μm. *b,* visualizing purinosome formation in hypoxic cells using EGFP-tagged ADSL. Fluorescent clusters can be observed in hypoxic cells. The DAPI-stained nuclei are shown in *blue*. *Scale bar* = 25 μm. *c,* quantifying the number of purinosome-containing cells transfected with FGAMS-mCherry in normoxia, after 24 h in hypoxia in purine-rich medium, and normoxia with purine-depleted medium (*purine -ve*). Data shown are *n* = 3, mean ± S.E., total number of cells counted are shown in *parentheses*. *d,* quantifying the number of purinosome-containing cells transfected with ADSL-EGFP in normoxia and hypoxia (24 h) in purine-rich medium. Data shown are *n* = 3, mean ± S.E., total number of cells counted are shown in *parentheses*. *e*, determining the number of clusters per cell and their average diameter in cells transfected with FGAMS-mCherry (*red*) or ADSL-EGFP (*green*). Total number of cells assessed is 157. *f*, the association of endogenous FGAMS and ADSL in hypoxia as measured by PLA (*red spots*; exemplar shown with a *white arrow*), with DAPI-stained nuclei in *blue*. Normoxic cells show no PLA signal, PLA signal is observed in cells incubated in hypoxia (in purine-rich media), or in purine-depleted media (in normoxia). *Scale bar* = 25 μm, uncropped images are deposited in the raw data files. *g,* quantification of cells showing a positive PLA signal in normoxia, hypoxia in purine-rich medium, and normoxia in purine-depleted media (*purine -ve*). Data shown are *n* = 7, mean ± S.E., total number of cells counted are shown in *parentheses*.

We first investigated the possibility that hypoxia enhances the assembly of the multienzyme purinosome complex. HeLa cells were transfected with a construct encoding formylglycinamidine ribonucleotide synthase (FGAMS), which catalyzes step 4 of *de novo* purine biosynthesis, as a fusion with the fluorescent protein mCherry (FGAMS-mCherry). These cells were cultured for 24 h in hypoxia (1% environmental oxygen) in the presence of purines (so far, purinosome formation had only been observed in cells cultured in purine-depleted media), and the degree of purinosome formation, as noted by enzyme clustering into distinct punctate structures, was assessed by fluorescence microscopy (2, 22, 23). We observed 40% of cells showing the clustering of FGAMS-mCherry in response to hypoxia compared with the 19% of cells in normoxia (Fig. 1, *a* and *c*). This was comparable with the ∼2-fold increase in purinosome-containing cells observed when cells were cultured in purine-depleted medium in normoxia (Fig. 1*c*). This experiment was repeated with ADSL, which catalyzes step 8 of *de novo* purine biosynthesis, tagged with GFP (ADSL-EGFP). Similar to that of FGAMS-mCherry, we observed a 2-fold increase in cells showing clustering of ADSL-EGFP in hypoxia (Fig. 1, *b* and *d*). Colocalization analysis between FGAMS-mCherry and ADSL-EGFP by confocal microscopy showed a high degree of colocalization in hypoxia (Pearson's coefficient of 0.83) and was validated by three-dimensional volume reconstitution showing clear isolated peaks within the cytoplasm, supporting the notion that purinosomes form in response to hypoxia (Fig. S1, *a* and *b*).

The number of fluorescent clusters per cell was next measured using HeLa cells transfected with either FGAMS-mCherry or ADSL-EGFP (24). We found that the number of clusters per cell varied between 6 and 220 with the median being 42 clusters per cell (Fig. 1*e*). In addition, we analyzed the diameter of the clusters and found that these ranged from 0.6 to 1.9 μm, with an average diameter of 0.96 ± 0.25 μm (Fig. 1*e*). To ensure that our observation of hypoxia-mediated purinosome formation was not cell line-specific, we transfected the plasmid encoding FGAMS-mCherry in MDA-MB-231 cells and observed an increase in purinosome-positive cells in hypoxia similar to that observed in HeLa (Fig. S2, *a* and *c*).

**Figure 2.**
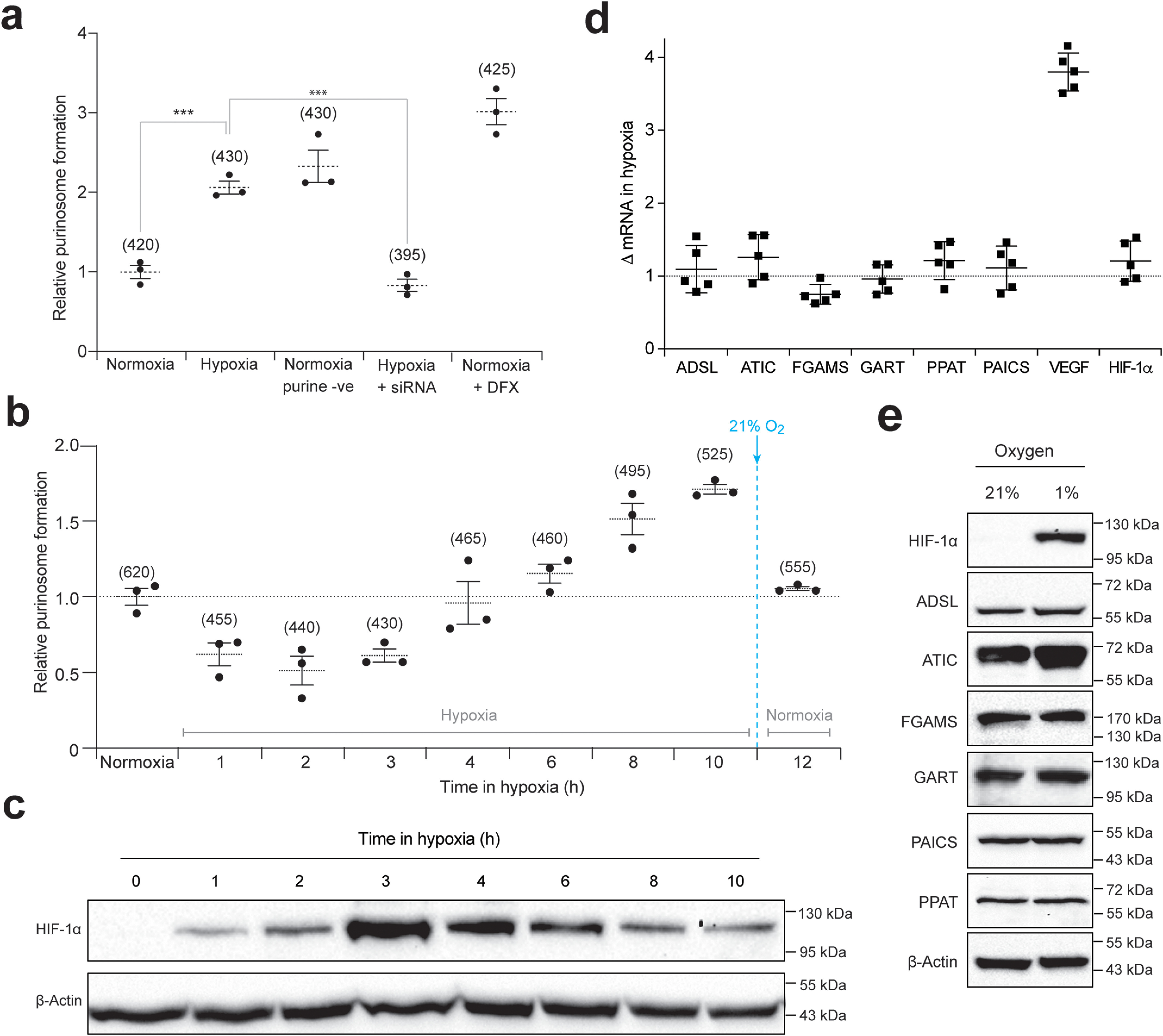
**The role of HIF-1 in purinosome formation.** *a,* quantifying the number of purinosome-containing cells in normoxia or hypoxia (24 h) in purine-rich medium and normoxia in purine-depleted medium (*purine -ve*), cells in hypoxia transfected with siRNA to HIF-1α (+ *siRNA*), and cells in purine-rich medium supplemented with DFX. Data shown are *n* = 3, mean ± S.E., total number of cells counted are shown in *parentheses*. *b,* time course of purinosome formation in hypoxia shows the number of purinosome-containing cells steadily increases after 3 h in hypoxia. Re-oxygenation of the samples after hypoxic incubation for 10 h reverts the number purinosome-containing cells back to normoxic levels. Data shown is *n* = 3, mean ± S.E., total number of cells counted are shown in *parentheses*. *c,* time course of HIF-1α stabilization in hypoxia shows maximum HIF-1α protein expression levels at 3 h in hypoxia, after which the HIF-1α expression decreases. The positions of molecular markers are shown for each blot; uncropped blots with overlaid markers are deposited in the raw data files. *d,* the effect of hypoxia on the transcription of purine biosynthesis enzymes measured by qPCR. Vascular endothelial growth factor (*VEGF*) and HIF-1α are controls. Data shown are *n* = 5, mean ± S.E. *e,* the effect of hypoxia on the protein expression levels of the purine biosynthetic enzymes. HIF-1α is stabilized in hypoxia as expected, and no significant increase in the purine enzymes was detected between normoxic (21% oxygen) and hypoxic (1% oxygen) growth conditions. The positions of molecular markers surrounding each band of interest are shown for each blot; uncropped blots with overlaid markers are deposited in the raw data files.

Hypoxia is known to induce oxidative stress (25), so we investigated whether these intracellular puncta were distinct from stress granules. HeLa cells were co-transfected with plasmids encoding FGAMS-mCherry and GFP-tagged RasGAP-associated endoribonuclease (GFP-G3BP), an intracellular marker for stress granules (3, 26), and colocalization was assessed by fluorescence microscopy. No overlap in the signals from these two markers was observed (Pearson's coefficient of −0.003), indicating that the observed clusters of FGAMS-mCherry in hypoxia are not associated with stress granules (Fig. S1*c*). In addition, we assessed whether hypoxia increases the number of stress granule-positive cells (Fig. S1*d*). We observed comparable numbers of cells containing stress granules in normoxia and hypoxia, indicating that the formation of the purinosome complexes in hypoxia is not a result of enhanced cellular stress. Previous reports note that the formation of the purinosome complex correlates to the cell cycle, reaching a maximum in G_1_ phase (22). We therefore quantified purinosome formation in the G_1_ phase of synchronized HeLa cells in hypoxia, however, we observed a similar ratio of purinosome containing cells in hypoxia (28%) as when using nonsynchronized cells (Fig. S1*e*).

To verify that hypoxia-induced purinosome formation occurs on the endogenous level, we developed a proximity ligation assay (PLA) to visualize and quantify the association of pathway enzymes, FGAMS and ADSL, within purinosomes (27). As this method probes endogenous proteins, any observed clustering of these enzymes cannot be a consequence of their transient overexpression nor due to interactions between the fluorescent protein tag(s). We observed 88% of hypoxic cells having a colocalization between FGAMS and ADSL, whereas few (10%) normoxic cells showed colocalization (Fig. 1, *f* and *g*). As a positive control, we confirmed an association of endogenous FGAMS and ADSL in 87% of normoxic cells cultured in purine-depleted medium (Fig. 1, *f* and *g*). To further support this, we assessed the colocalization of FGAMS and GART (a trifunctional protein that catalyses steps 2, 3, and 5 of the *de novo* purine biosynthetic pathway), and observed an increase in the number of PLA signal-positive cells in hypoxia (81%) compared with normoxia (4%) (Fig. S1, *f* and *g*), which is in line with the results observed for ADSL and FGAMS. These data suggest that a substantially higher subset of cells form purinosomes in hypoxia (and in purine-depleted normoxia) than observed when using fluorescently-tagged proteins (88 *versus* 40%). This may be a consequence of the need for multiple copies of a given fluorescently tagged proteins to be present in a purinosome cluster before it can be observed by fluorescent microscopy. Alternatively, the presence of the fluorescent protein may impede complete purinosome formation or be less favorable than the complexation of endogenous proteins. Together the above data demonstrates that purinosome assembly is up-regulated by hypoxia in HeLa cells.

### Deciphering the role of HIF-1 in hypoxia-mediated purinosome assembly

HIF-1 plays a central role in the cellular hypoxia response, and we therefore sought to assess whether activation of HIF-1 correlates with purinosome formation in hypoxia. HeLa cells transiently expressing FGAMS-EGFP were treated with HIF-1α siRNA prior to incubation in hypoxia for 24 h. Consistent with before, a 2-fold increase in purinosome formation was observed in hypoxic cells (Fig. 1*c* and Fig. 2*a*); however, in cells lacking HIF-1α, purinosome formation was not detected (Fig. 2*a*). The role of HIF-1 in this process was further probed using desferrioxamine (DFX), which chemically stabilizes HIF-1α in normoxia (28). If purinosome formation in hypoxia is mediated through HIF-1α stabilization, then DFX treatment will be sufficient to enhance purinosome formation in normoxia. We observed a ∼3-fold increase in purinosome formation in DFX-treated cells cultured in normoxia (Fig. 2*a*).

The isoform specificity of HIF-1 in promoting purinosome formation was probed using 786-O cells, which under hypoxic conditions use the HIF-2α isoform, instead of HIF-1α, and the HIF-2 transcription factor (29). We did not observe any hypoxia-driven increase in purinosome formation in these cells (Fig. S2, *b* and *c*). To ensure that our data were not a result of an inherent inability of 786-O cells to form purinosomes, we incubated these cells under normoxic purine-depleted conditions and observed a ∼6-fold increase in purinosome formation (Fig. S2*b*). This confirms the necessity of the HIF-1α isoform in driving purinosome formation in hypoxic cells, and this phenomenon is not replaceable with the HIF-2α isoform.

To further probe the link between HIF-1α stabilization in hypoxia and purinosome formation, we carried out a time course experiment. Purinosome assembly and HIF-1α levels were assessed in HeLa cells incubated under hypoxic conditions at intervals over a 10 h period. We observed a ∼50% reduction in the number of purinosome-containing cells within the first 2 h of hypoxia exposure followed by a steady increase where it eventually levelled off after 8-10 h (Fig. 2*b*, Video S1). During the same time course, HIF-1α protein levels showed the highest expression at 3 h and gradually decreased over time (Fig. 2*c*), consistent with prior reports (30, 31). However, this reduction in HIF-1α did not alter the number of cells containing purinosomes (Fig. 2*b*), presumably because the downstream effects of HIF-1α activation persist.

Purinosomes are highly dynamic structures that readily dissipate in response to high purine levels in culturing media (2). We therefore investigated whether purinosomes formed in hypoxic cells were similarly dynamic and could be disrupted by reoxygenation. After 10 h in hypoxia, cells were reincubated in normoxia for 2 h, and the number of purinosome containing cells counted. Upon reoxygenation, the ratio of purinosome-containing cells had reverted back to normoxic levels (Fig. 2*b*), demonstrating the reversible and transient nature of this multienzyme complex.

Cellular adaptation to hypoxia is driven by HIF-1–mediated transcription of multiple genes, several of which encode metabolic enzymes (14, 32). We therefore assessed whether the observed increase in purinosome formation in hypoxia is a result of an up-regulation in gene or protein expression of its enzymes. The expression levels of the six genes encoding the enzymes in *de novo* purine biosynthesis remained unchanged after exposure to hypoxia for 24 h (Fig. 2*d*). Protein expression by immunoblotting also showed no changes in the levels of the purinosome enzymes except for a slight increase in intracellular ATIC protein levels (Fig. 2*e*). These data eliminated the possibility that the increase in purinosome formation under hypoxic conditions is caused by an up-regulation in transcription or translation of the enzymes within the *de novo* purine biosynthesis pathway.

### Inhibition of hypoxia-mediated purinosome formation

It has been previously demonstrated that the formation of the purinosome complex is disrupted in cells lacking one of the enzymes in *de novo* purine biosynthesis or by introducing loss-of-function mutations in ADSL and/or ATIC (7, 33).

**Figure 3.**
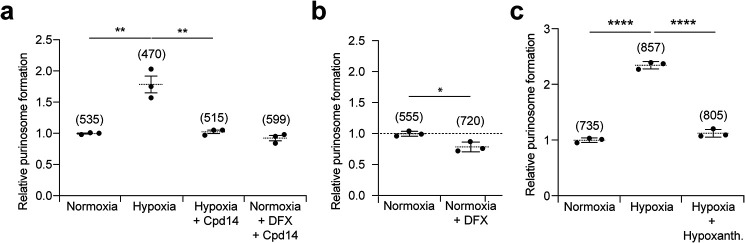
**Inhibiting purinosome formation in hypoxic cells.** *a,* the effect of Cpd14 inhibitor of ATIC on purinosome formation in hypoxia and normoxic cells treated with 100 μm DFX (24 h). In both conditions, Cpd14 at 250 μm prevented the hypoxia-induced purinosome formation. Data shown are *n* = 3, mean ± S.E., total number of cells counted are shown in *parentheses*. *b,* visualizing purinosome formation in ATIC knockout (*KO*) HeLa cells using FGAMS-EGFP in normoxia in purine-rich media untreated and in normoxia in purine-rich media supplemented with 100 μm DFX (24 h). In ATIC KO cells, addition of DFX did not induce purinosome formation. Data shown are *n* = 3, mean ± S.E., total number of cells counted are shown in *parentheses*. *c,* quantifying the number of cells with purinosomes marked with FGAMS-mCherry in purine-rich media in normoxia and hypoxia as well as purine-rich media supplemented with 60 μm hypoxanthine in hypoxia. Treatment of HeLa with hypoxanthine in hypoxia reverted the purinosome formation to normoxic levels. Data shown are *n* = 3, mean ± S.E., total number of cells counted are shown in *parentheses*.

ATIC is a homodimeric enzyme, and we have previously reported a small molecule that disrupts its activity by inhibiting dimerization, named Compound 14 (Cpd14) (34, 35). We used Cpd14 to probe whether disruption of ATIC homodimerization leads to inhibition of purinosome formation in hypoxia. HeLa cells were transfected with a plasmid encoding FGAMS-EGFP and treated with Cpd14 for 24 h in purine-rich media in hypoxia or normoxia supplemented with DFX. In both cases, treatment with Cpd14 prevented hypoxia- and DFX-induced purinosome formation (Fig. 3*a*). Similarly, purinosome formation was assessed in an ATIC knockout HeLa cell line (33) under normoxic growth conditions in the presence or absence of DFX (used instead of hypoxia to allow a side-by-side comparison). No increase in purinosome formation was observed in cells incubated with DFX (Fig. 3*b*). The absence of ATIC or disruption of its dimerization via Cpd14 was sufficient to prevent purinosome formation in hypoxia.

Purinosome formation has been shown to coincide with an up-regulation of *de novo* purine biosynthesis (5). We therefore probed whether stimulating the salvage pathway by adding hypoxanthine (the precursor of IMP in purine salvage) to the cell culture medium (36) would lead to disruption of purinosome formation. Hypoxanthine-treated HeLa cells were incubated in hypoxia and purinosome formation was assessed as before. We observed no increase in purinosome formation upon hypoxic incubation in these cells, suggesting that up-regulation of purine salvage might lead to disruption of purinosome formation (Fig. 3*c*). Together, these data further demonstrate that the clusters observed in hypoxia behave as purinosomes and that their formation can be disrupted by targeting enzymes of purine biosynthesis.

### Uncovering cellular processes affecting hypoxia-mediated purinosome assembly

**Figure 4.**
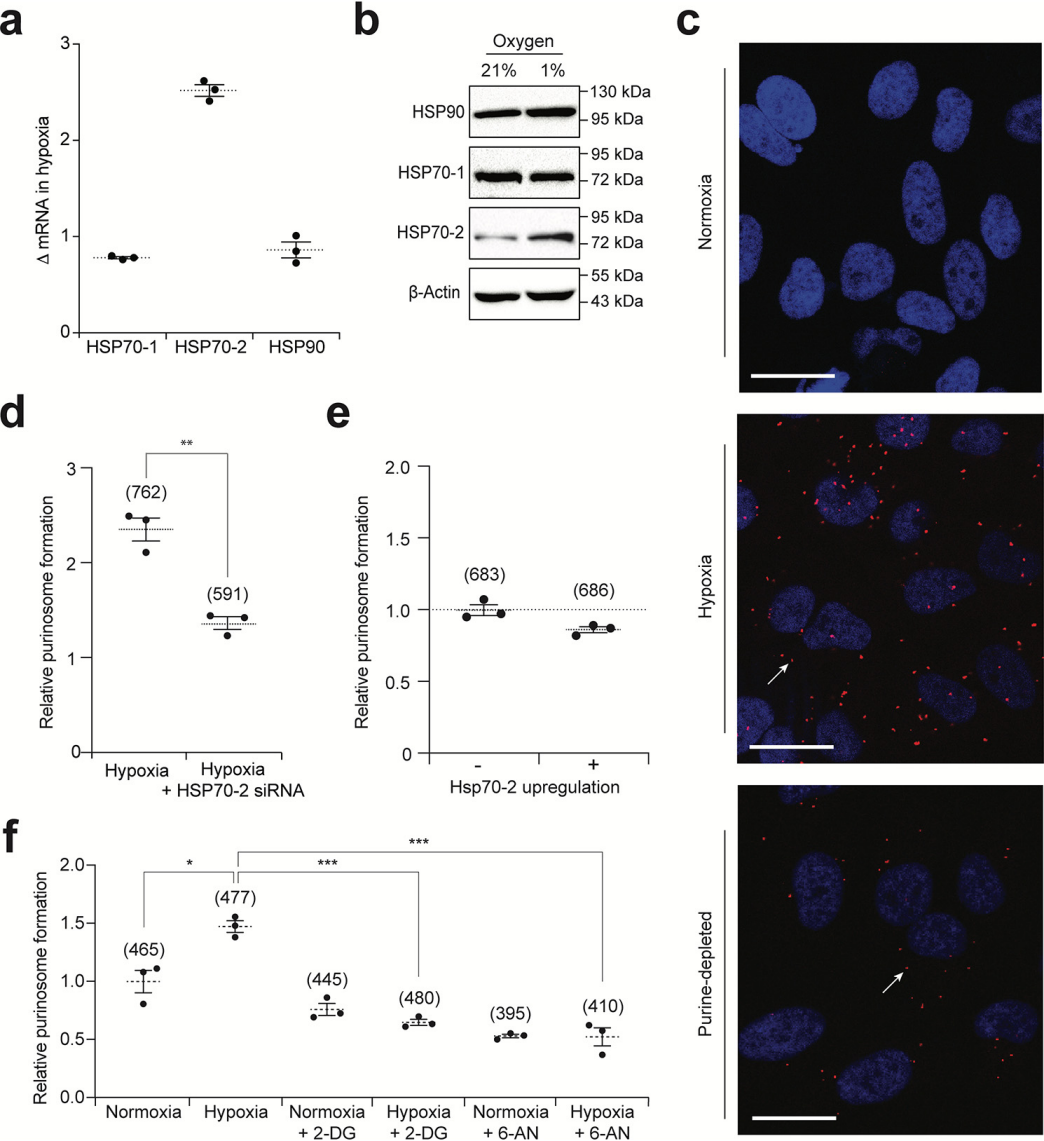
**Probing the mechanism of purinosome formation in hypoxia.** *a*, The effect of hypoxia (24 h) on the transcription of *HSP70-2*, *HSP70-1,* and *HSP90* measured by qPCR. Data shown are *n* = 3, mean ± S.E. *b,* the effect of hypoxia (24 h) on the protein expression of HSP90, HSP70-1, and HSP70-2. *c,* the association of endogenous ADSL and HSP70-2 in normoxic and hypoxic cells (both in purine-rich medium) or normoxic cells in purine-depleted medium (24 h). The *red spots* (PLA signal, exemplar shown by *white arrow*) arise from interacting proteins, whereas the DAPI-stained nuclei are in *blue*. *Scale bar* = 25 μm, uncropped images are deposited in the raw data files. *d,* quantifying the number of cells with purinosomes marked with FGAMS-mCherry in hypoxia (24 h) with (+ *siRNA*) or without siRNA to HSP70–2 (− *siRNA*). Data shown are *n* = 3, mean ± S.E., total number of cells counted are shown in *parentheses*. *e*, the effect of HSP70–2 overexpression on purinosome formation in cells using FGAMS-mCherry in normoxia (24 h). Data shown are *n* = 3, mean ± S.E., total number of cells counted are shown in *parentheses*. *f*, the effect of the glycolysis inhibitor 2-DG and the PPP inhibitor 6-AN on purinosome formation in normoxia and hypoxia (6 h). Both molecules revert the number of purinosome-containing cells in hypoxia to normoxic levels, with no effect on normoxic cells. Data shown are *n* = 3, mean ± S.E., total number of cells counted are shown in *parentheses*.

Having demonstrated the central role of HIF-1α in driving purinosome assembly in hypoxia, we aimed to unravel the mechanistic basis of this phenomenon. One hypothesis was formed around HIF-1 affecting heat shock protein (HSP) levels, as HSP90 and isoform 1 of HSP70 (HSP70–1) have both been reported to be critical for purinosome assembly in purine-deficient media (26, 37). We began by using qPCR to measure the effect of hypoxia on the transcription of *HSP90*, *HSP70-1*, and isoform 2 of HSP70 (*HSP70*–*2*). Hypoxia did not alter the transcription of *HSP90* and *HSP70-1*, but in line with previous reports, we observed a ∼2.5-fold increase in *HSP70-2* in hypoxic cells (Fig. 4*a*) (38). The increase in *HSP70*–*2* transcription did not occur when cells were treated with siRNA to HIF-1α prior to incubation in hypoxia (Fig. S3*a*). Western immunoblotting showed that this correlated to an increase in HSP70–2 protein in hypoxia, while HSP70–1 and HSP90 levels remained unchanged (Fig. 4*b*). These data suggest that the HIF-1–mediated increase in HSP70–2 may play a role in forming and/or stabilizing purinosome complexes in hypoxia.

We therefore probed for an interaction between HSP70–2 and ADSL by PLA in cells incubated in normoxia, hypoxia, and purine-depleted normoxia. ADSL is not a known client of HSP90 or HSP70; therefore, any detected association would be due to the proximity of these proteins in a purinosome (37). PLA signals between ADSL and HSP70–2 were observed in 92% of cells incubated in hypoxia and only 1% of cells in normoxia, indicating an increase in the association of these two proteins in response to hypoxia (Fig. 4*c*). Interestingly, only 29% of the cells cultured in purine-depleted conditions showed interaction of HSP70–2 and ADSL (Fig. S3*b*) suggest this isoform of HSP70 plays a less important role in stabilizing purinosomes under depleted conditions. The necessity of HSP70–2 for purinosome formation in hypoxic cells was assessed using cells treated with siRNA to HSP70–2 prior to incubation in hypoxia for 24 h. Knockdown of HSP70–2 resulted in a significant reduction in the number of purinosome-containing cells (Fig. 4*d*). Although this result demonstrates that HSP70–2 is sufficient for purinosome formation, it is not clear whether up-regulation of HSP70–2 in hypoxia drives purinosome assembly or serves to stabilize purinosomes already formed by another mechanism. We investigated this by overexpressing HSP70–2 in HeLa cells (Fig. S3*c*), and assessing the effect on the formation of intracellular FGAMS-mCherry puncta. If the HIF-1–mediated overexpression of HSP70–2 is the driver of purinosome assembly in hypoxia, then an increase in the number of purinosome-containing cells would be observed when HSP70–2 is overexpressed in normoxic cells. However, we did not observe a difference in purinosome formation in cells overexpressing HSP70–2 compared with cells transfected with the FGAMS-mCherry plasmid alone (Fig. 4*e*). These data demonstrate that HSP70–2 up-regulation does not lead to purinosome formation in cells, and the increase in HSP70–2 expression observed in hypoxia likely serves to stabilize purinosomes.

A second hypothesis was evaluated to determine whether purinosome assembly in hypoxia is driven by the HIF-1–mediated increase in the metabolites of glycolysis and the PPP that form the starting material and substrates for the *de novo* purine biosynthesis. If purinosome formation in hypoxia is driven by an increase in the metabolites of glycolysis and/or the PPP, then the HIF-1–mediated increase in purinosome formation will be ablated in those inhibitor-treated cells. To test this hypothesis, cells were treated with either 2-deoxyglucose (an inhibitor of glycolysis) or 6-aminonicotinamide (an inhibitor of the PPP) prior to incubation in hypoxia for 6 h, and the degree to which purinosome formation was perturbed, was determined (39–41). We observed that treatment with either the glycolytic inhibitor or the PPP inhibitor reduced purinosome formation in hypoxic cells to levels observed in normoxic cells (Fig. 4*f*). To further support this, we knocked down glucose-6-phosphate dehydrogenase (Glc-6-PD), the first enzyme of the PPP that branches glycolysis to the PPP, with shRNA. A reduction in the number of purinosome-positive hypoxic cells treated with Glc-6-PD shRNA was observed when compared with untreated hypoxic cells (Fig. S3*d*). No significant decrease in the number of purinosome-positive cells was observed upon treatment with the scrambled shRNA. These results indicate that the HIF-1–mediated up-regulation of glycolysis and the PPP, as well as the subsequent increase in associated metabolites in hypoxia is required for purinosome assembly in hypoxic cells.

### Colocalization of purinosomes with mitochondria in hypoxia

**Figure 5.**
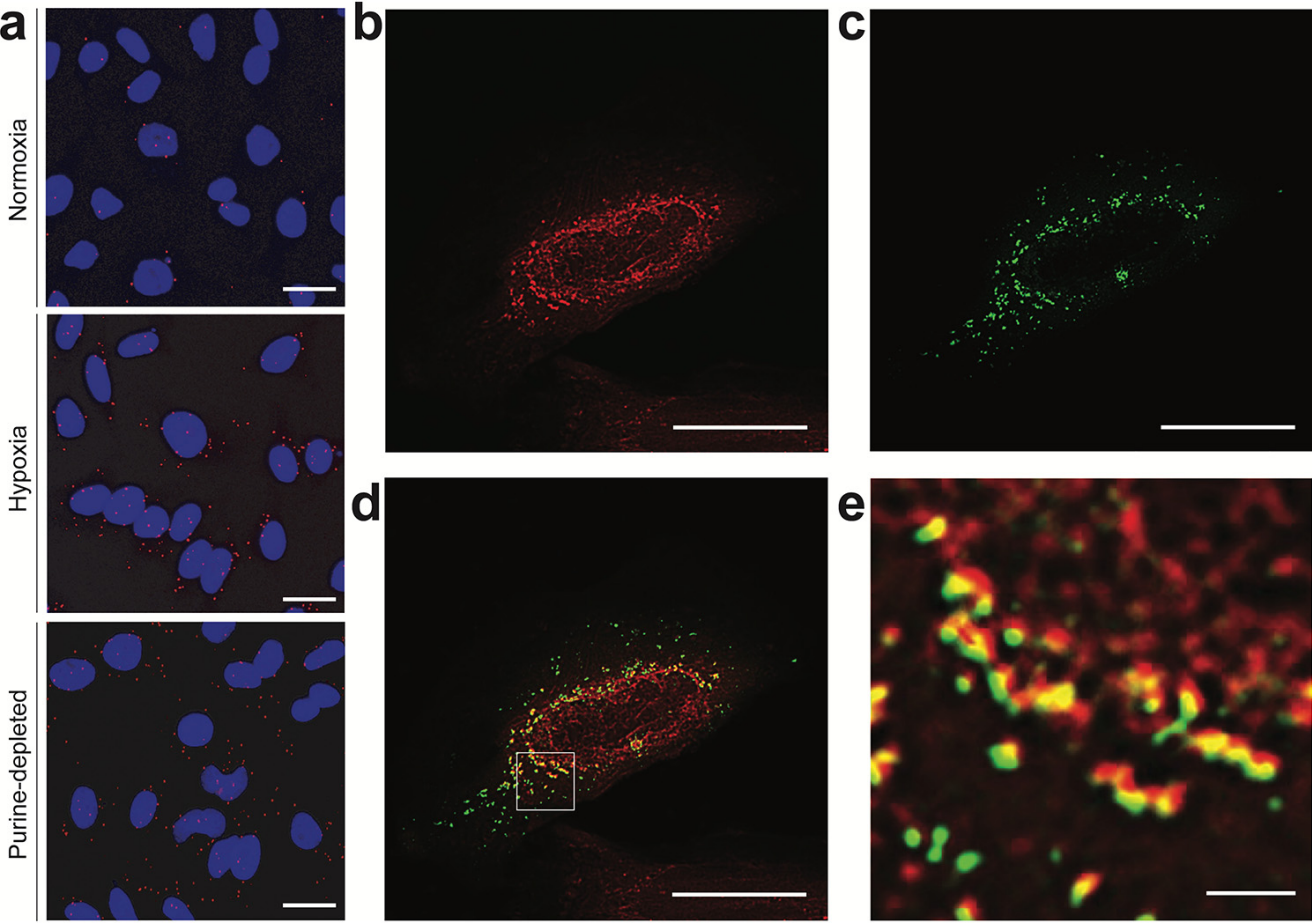
**The purinosome complex colocalizes with the mitochondria in hypoxia.** *a,* proximity ligation assay was carried out between FGAMS and an outer mitochondrial membrane protein, TOM20. Increased colocalization of the two proteins was found in hypoxia and normoxia in purine-depleted conditions. Quantification data are shown in Fig. S4. *Scale bar* = 25 μm. *b–e*, colocalization analysis between the mitochondria and purinosomes in hypoxic HeLa cells using confocal imaging coupled to SRRF analysis. *Scale bar* = 25 μm. *b,* MitoTracker Deep Red staining of mitochondria. *c,* FGAMS-EGFP–labeled purinosomes. *d,* merged image of *b* and *c*. *e,* 8.3-fold magnification of ROI presented as a *white square* in *d*. *Scale bar* = 2 μm.

Up-regulation of glycolysis in response to hypoxia can also feed into the serine biosynthetic pathway and one-carbon metabolism (42). These processes have been tied to *de novo* purine biosynthesis as the one-carbon unit generated from serine and tetrahydrofolate (THF) becomes integrated into the essential cofactor, 10-formyl-THF, for the activity of two purinosome enzymes, GART and ATIC. The process of generating these one-carbon units in HeLa cells is predominately done in the mitochondria (43). Previous reports revealed that purinosomes in purine-depleted conditions colocalized with mitochondria and hypothesized to transfer the necessary metabolites to increase purine production (8, 44). We therefore assessed the colocalization of purinosomes with mitochondria in hypoxia. We observed the proximity of the purinosome complex (FGAMS-EGFP) and the mitochondria (MitoTracker Deep Red) in hypoxic HeLa cells by confocal microscopy coupled to super-resolution radial fluctuations (SRRF) analysis (45). When merging the two channels (Fig. 5, *b* and *c*), clear colocalization between the two fluorophores was observed throughout the cell (Fig. 5*d*) with a Pearson's colocalization coefficient of 0.525, indicating a significant colocalization between purinosomes and mitochondria in hypoxic HeLa cells. We used a region of interest (ROI) from the merged image (Fig. 5*d*, *white square*) and magnified it 8.3-fold to obtain a more detailed view (Fig. 5*e*). This ROI revealed the presence of purinosomes (*green channel*) located on the mitochondria (*red channel*), suggesting that the complexes sit on the mitochondrial network, as previously observed for purinosomes induced using purine-depleted conditions (44). We validated this result on the endogenous level by probing the proximity between FGAMS and TOM20, a protein located on the outer mitochondrial membrane by PLA. We found very little PLA signal in cells in normoxia (8%), whereas hypoxic conditions induced a substantial increase in the number of cells containing PLA signals (95%) (Fig. 5*a*, Fig. S4), suggesting increased colocalization between the purinosome and the mitochondria in hypoxic HeLa cells. As a positive control, we also assessed the colocalization of these proteins in purine-depleted conditions. PLA signals were observed in 96% of these cells, in line with the data for hypoxic cells (Fig. 5*a*, Fig. S4). Together, these data support that the purinosome is colocalized with mitochondria and open up the question whether the purinosome-mitochondria colocalization contributes to enhance purine production in hypoxia.

### Assessing the effect of hypoxia-induced purinosomes on purine biosynthesis

**Figure 6.**
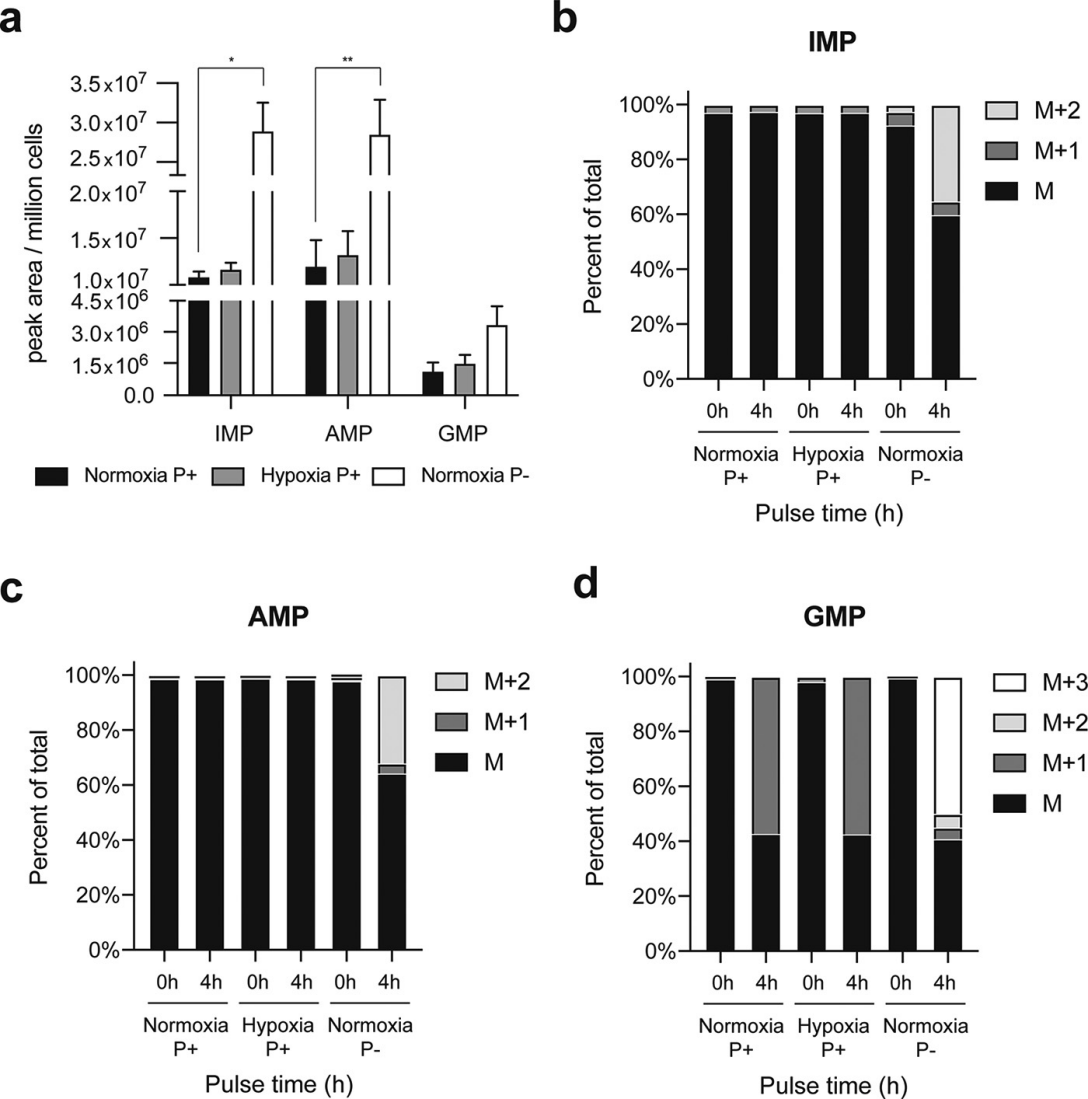
**Incorporation of [^15^N]glutamine in purine nucleotide monophosphates.** *a,* total abundance of IMP, AMP, and GMP was determined in normoxia and hypoxia (*P*+) and in normoxia purine-depleted (*P*−) after 16 h of hypoxic incubation. *b,* incorporation of [^15^N]glutamine into IMP. [^15^N]Glutamine incorporation into IMP was not detected in cells cultured in hypoxia in purine-rich media (*P*+) but was observed in those cultured in normoxia in purine-depleted media (*P*−) suggesting an up-regulation of the *de novo* pathway only in normoxia P−. *c,* incorporation of [^15^N]glutamine into AMP. [^15^N]Glutamine incorporation into AMP was not detected in hypoxia P+ but was observed in normoxia P−, suggesting an up-regulation of the *de novo* pathway only in normoxia P−. *d,* incorporation of [^15^N]glutamine into GMP. M+3 GMP was not detected in hypoxia P+ but was observed in normoxia P− suggesting an up-regulation of the *de novo* pathway only in normoxia P−. Data shown are *n* = 3, mean ± S.E.

A previous report has correlated purinosome formation in purine-deprived cells with an increase in *de novo* purine biosynthesis (5). We first determined the total abundance of IMP, AMP, and GMP in HeLa cells cultured in normoxia and hypoxia in the presence of purines, as well as in normoxia in the absence of purines. There was no difference in total IMP, AMP, and GMP abundance between HeLa cells cultured in hypoxia compared with those cultured in normoxia; whereas, cells that were cultured under purine-depleted normoxic conditions showed a significant increase in the total IMP and AMP levels (Fig. 6*a*), consistent with previous reports (5). To determine whether the *de novo* pathway was utilized in hypoxia, ^15^N-labeled glutamine was incubated with preconditioned HeLa cells for 4 h and its incorporation into newly synthesized purines was evaluated. Label incorporation studies were also performed in normoxic HeLa cells in the presence and absence of purines (positive control). Isotope incorporation into IMP and AMP was observed in cells cultured under purine-depleted conditions as indicated by the presence of M+2 species (Fig. 6, *b* and *c*). However, no label incorporation was observed in the other conditions tested, suggesting that the *de novo* synthesis of purine monophosphates is not up-regulated in hypoxia (Fig. 6). This conclusion was verified by looking at the isotope incorporation into GMP. If GMP is synthesized through the *de novo* pathway, then an M+3 species is to be expected; however, if GMP is synthesized from the salvage pathway (*i.e.* unlabeled hypoxanthine recycled into IMP), then only an M+1 species would be observed. Please note that the conversion of IMP into GMP requires one molecule of glutamine. We only observed the presence of an M+1 species in purine-supplemented normoxic and hypoxic conditions (Fig. 6*d*). Based from this study, we verified that under our hypoxic experimental growth conditions, purine monophosphates are not being synthesized by the *de novo* pathway.

To determine whether this preferential synthesis via the salvage pathway was the result of an increased expression of the salvage enzymes, we assessed the effect of hypoxia on the mRNA and protein levels of the salvage pathway enzymes APRT and HPRT in hypoxic HeLa cells. We observed no effect from hypoxia on gene expression and protein levels of APRT or HPRT in our experiments (Fig. S5, *a* and *b*, respectively), suggesting that the salvage purine synthesis does not result from an increase in salvage enzymes expression in hypoxia. In addition, we assessed whether the salvage enzymes were part of the purinosome complex in hypoxia. To probe this, we performed PLA between ADSL and APRT (Fig. S5*c*), as well as ADSL and HPRT (Fig. S5*d*). Both interactions were found to occur in normoxia, and no increase in the occurrence of these interactions was observed in hypoxia. These results suggest that the salvage enzymes do not associate with the purinosome in hypoxic conditions.

### Probing the effect of hypoxia on substrate production for de novo purine biosynthesis

**Figure 7.**
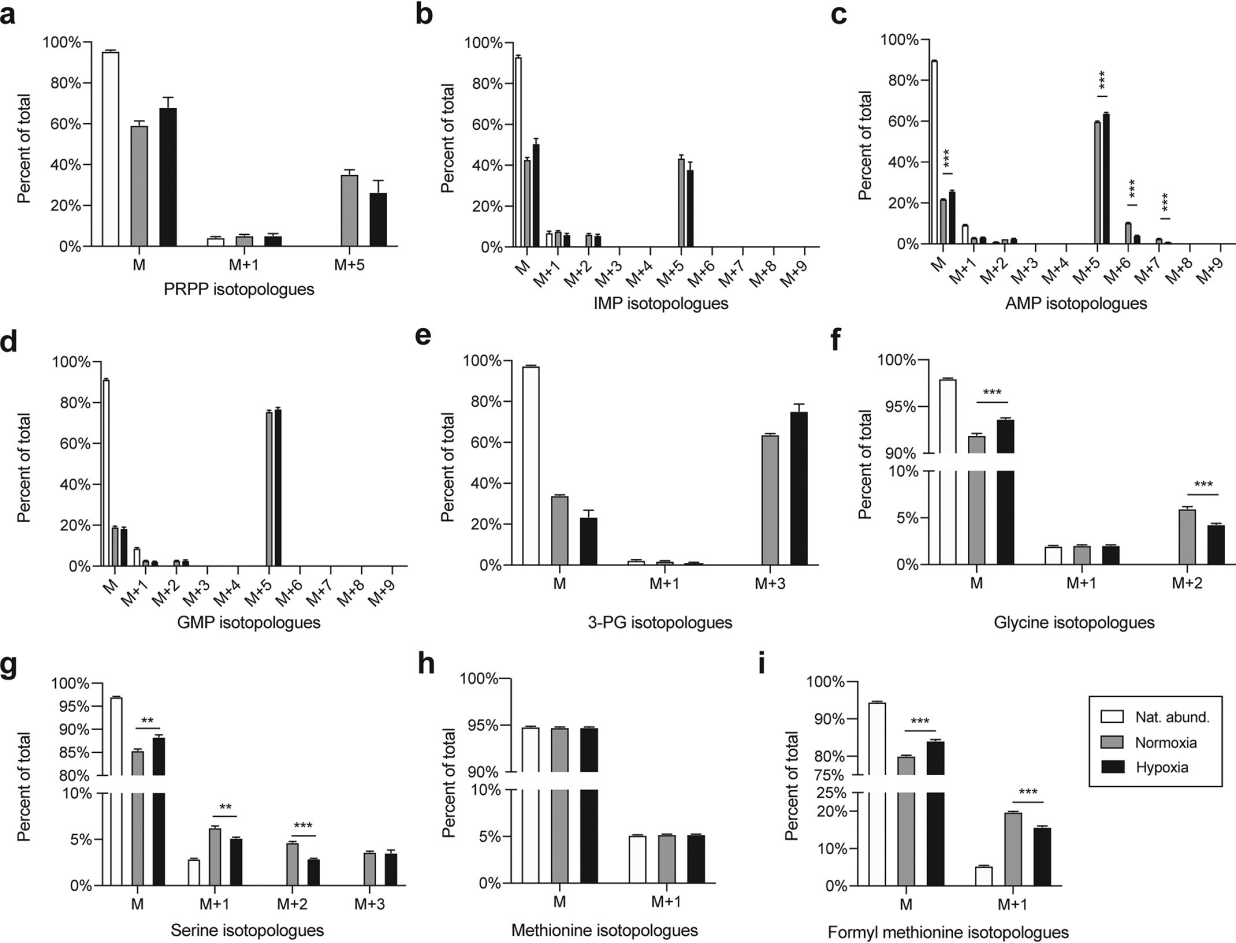
**Incorporation of [^13^C_6_]glucose into purine nucleotide monophosphates and metabolites from associated pathways.** *a–d*, incorporation of isotope labeled from [^13^C_6_]glucose traced into *de novo* purine biosynthesis metabolites: *a,* PRPP; *b,* IMP; *c,* AMP; *d,* GMP. *e–j*, incorporation of isotope labeled from [^13^C_6_]glucose traced into metabolites from pathways associated with *de novo* purine biosynthesis: *e,* 3-PG; *f,* glycine; *g,* serine; *h,* methionine; and *i,* formylmethionine. Data shown are *n* = 4, mean ± S.E.

The above data demonstrated that despite the formation of purinosomes, the *de novo* purine biosynthetic pathway is not active in hypoxia. We then asked whether changes in substrate or cofactor generation was the cause of this observation. The *de novo* purine biosynthetic pathway relies on the PPP and mitochondrial one-carbon metabolic pathways to generate the PRPP and glycine substrates and the formate needed for cofactor biosynthesis (21). To investigate whether this was occurring, [^13^C_6_]glucose was introduced to hypoxic or normoxic HeLa cells for 24 h and isotope incorporation was determined for selected metabolites. The main substrate of both *de novo* and salvage pathways for purine synthesis, PRPP, showed an M+5 species both in normoxia and hypoxia, without any significant difference in the levels of the newly synthesized metabolite between the two conditions (Fig. 7*a*).

Furthermore, [^13^C_6_]glucose was processed through glycolysis leading to the formation of [^13^C_3_]phosphoglycerate ([^13^C_3_]3-PG), the first substrate of serine biosynthesis. The labeled species can then be converted into [^13^C_3_]serine and further traced into the products of one-carbon metabolism: [^13^C_2_]glycine and [^13^C]formate. We anticipate that these newly synthesized isotope-labeled molecules will be integrated into purine monophosphates if the *de novo* pathway was utilizing these metabolites. The glycolytic intermediate, 3-PG, was found to display an M+3 isotopologue both in normoxic and hypoxic conditions with a slight increase in hypoxia, consistent with an increased rate of glycolysis in these conditions (Fig. 7*e*). The presence of [^13^C_3_]serine (M+3) was observed in both normoxic and hypoxic conditions with no significant difference, indicating that [^13^C_3_]serine is synthesized *de novo* from 3-PG under both conditions (Fig. 7*g*). Interestingly, a decrease in the levels of [^13^C_2_]glycine (M+2) was observed in hypoxic conditions, which indicates a decreased conversion of [^13^C_3_]serine into [^13^C_2_]glycine (Fig. 7*f*). Furthermore, the presence of decreased levels of [^13^C_2_]serine reveals a decreased back-conversion of [^13^C_2_]glycine into [^13^C_2_]serine, likely as a result of decreased [^13^C_2_]glycine availability.

The observed reduction in serine to glycine conversion is likely due to down-regulation of a mitochondrial one-carbon metabolism, the predominant origin of one-carbon units for HeLa cells (43). To determine the impact of hypoxia upon the activity of the mitochondrial branch of the one-carbon metabolism, we analyzed the [^13^ C]glucose label incorporation into formylmethionine (fMet), a metabolite that uses mitochondrial 10-formyl-THF as cofactor in its synthesis. A decrease in isotope incorporation into fMet in hypoxia was observed, suggesting a decreased activity of the mitochondrial one-carbon metabolism in hypoxic HeLa cells (Fig. 7*i*). This observation was consistent with our observed decrease in serine to glycine conversion (Fig. 7*f*). Together, these data suggest that the activity of the mitochondrial one-carbon metabolism is down-regulated under hypoxic conditions, thus limiting the availability of cofactors required for the *de novo* purine biosynthesis. This lack of cofactors might be the reason why no increase in *de novo* purine biosynthesis is observed in hypoxia, despite the formation of purinosomes.

## Discussion

Multienzyme metabolic complexes have long been proposed (46); however, relatively little is known about physiological conditions that trigger their formation. One such example is the purinosome, which has been extensively studied in cells maintained in purine-depleted media and hypothesized to up-regulate purine biosynthesis (4, 5). Our studies began with an observation that purinosome formation is enhanced in hypoxic cells in the presence of purines. Here, we sought to fully characterize and decipher what drives complexation in hypoxia. Our results indicate a critical, isoform-specific role for the HIF-1 transcription factor in up-regulating purinosomes in hypoxia. However, this observation is not due to a HIF-1–mediated increase in the transcription or translation of the enzymes of *de novo* purine biosynthesis. We found that the increase in flux through glycolysis and PPP under hypoxic conditions is critical for the assembly of purine biosynthetic enzymes into purinosomes. We also observed that the HSP70–2 chaperone associates with the purinosome complex in hypoxia but was not sufficient to cause its assembly in normoxia. The process of purinosome formation in hypoxia was further found to be reversible upon reoxygenation of cells and could be prevented by either knocking down ATIC or using a small molecule inhibitor of ATIC homodimerization. These data demonstrate that the puncta observed in our experiments are purinosomes whose assembly may be disrupted by removing or disrupting the protein–protein interaction of a single enzyme from the associated pathway. Although our studies were focused on studying purinosome formation in hypoxia using HeLa cells, other cell lines tested such as MDA-MB-231 triple-negative breast cancer cells showed a similar phenotype to the hypoxia response.

Our observation of increased purinosomes formation in hypoxia led to the question of whether this phenomenon leads to an increase in purine production through the *de novo* pathway as previously observed for HeLa cells cultured in purine-deficient media. Our isotope incorporation studies suggest that diversions in glycolytic flux feeds into the pentose phosphate and the serine biosynthetic pathways to provide enough PRPP and serine to sustain purine production; however, no increase in *de novo* purine biosynthesis was observed. Rather, the nucleotide monophosphates are synthesized predominantly via the salvage pathway under hypoxia. Decreased metabolite flux through the *de novo* purine biosynthetic pathway, despite increased purinosome formation, is likely due to a down-regulation in one-carbon metabolism. Previous studies report that myc drives the up-regulation of the mitochondrial enzymes of one-carbon metabolism in hypoxia (47), however, c-myc levels have been shown to be significantly reduced in hypoxic HeLa cells (48). This provides a possible explanation for the absence of increased *de novo* purine biosynthesis in hypoxic HeLa cells, despite increased purinosome formation.

Overall, this study expands our understanding of purinosome formation and reveals that the biochemical processes that drive this process are not solely dependent on substrate or cofactor availability. Previous studies have shown that purinosome formation is associated with post-translational modifications such as phosphorylation (49), possibly as a consequence of activated AKT/mTOR and/or G protein–coupled receptor-mediated signaling pathways (44, 50). Exploring the similarities in the genetic reprogramming and signaling events between hypoxia and activation of *de novo* purine biosynthesis is expected to provide further insights into the molecular mechanism by which the enzyme of purine biosynthesis organize into purinosomes. Our future investigations are focused on probing the role of myc on purinosome function in hypoxia in a number of cell lines, including adipocytes where an increase in *de novo* purine biosynthesis is observed under hypoxia (51).

## Experimental procedures

### Reagents

All chemicals, including DFX (D9533), 2-deoxyglucose (2-DG) (D6134), 6-aminonicotinamide (6-AN) (A68203), and hypoxanthine (H9636), were purchased from Sigma-Aldrich. Cpd14 was synthesized as previously described (35). Plasmids hFGAMS-EGFP, hFGAMS-mCherry, hADSL-EGFP, and G3BP-GFP constructs were used in this study, as previously reported (2, 26). pcDNA5/FRT/TO HSPA2 was a gift from Harm Kampinga (Addgene plasmids number 19458). siRNA targeting HIF-1α (S6539), HSP90 (121532), HSP70 isoform 1 (145248), and HSP70 isoform 2 (145384) were purchased from Thermo Fisher Scientific. Antibodies targeting ADSL (NBP2-03107), APRT (NBP1-89519), ATIC (NBP2-01941), FGAMS (NBP1-84691), GART (H00002618-M01), HPRT (NBP1-33527), PAICS (NBP2-02817), PPAT (NBP2-02056), HIF-1α (NB100-449), HSP70 isoform 1 (NBP2-46806), and HSP70 isoform 2 (NBP1-86185) were purchased from Novus Biologicals. HSP90 antibody (Ab13492) and TOM20 antibody (Ab56783) was purchased from Abcam. Anti-β-actin (A3854) antibody was purchased from Sigma. Secondary anti-mouse (AP130P) and anti-rabbit (7074P2) HRP-conjugated antibodies were purchased from Thermo Fisher Scientific and Merck Millipore, respectively. All qPCR reagents and probes were purchased from Thermo Fisher Scientific: TaqMan Universal Master Mix (4369016), TaqMan gene expression assays 18S (Hs99999901_s1), β-actin (Hs99999903_m1), ADSL (Hs01075808_m1), APRT (Hs00356991_m1), ATIC (Hs00269671_m1), FGAMS (Hs00389822_m1), GART (Hs00894582_m1), HPRT (Hs02800695_m1), PAICS (Hs00935017_gH), PPAT (Hs00601264_m1), HIF-1α (Hs00153153_m1), HSP90AA (Hs00743767_sH), HSP70 isoform 1 (Hs00359163_s1), HSP70 isoform 2 (Hs00745797_s1), and VEGF (Hs00900055_m1). Previously reported sequences of Glc-6-PD short hairpin RNA (shRNA) (GTCGGATACACACATATTCA) and scramble Glc-6-PD shRNA (GCTCAACGGTCAACATATTA) were cloned into psiRNA-hH1-GFP-zeo plasmids (Invivogen) (52).

### Cell culture

HeLa, MDA-MB-231, and 786-O cells were purchased from American Type Culture Collection (ATCC). ATIC knock-out HeLa (ATIC KO) cells were a gift from Marie Zikanova and Veronika Baresova (Charles University and General University Hospital, Prague). HeLa, MDA-MB-231, and 786-O were cultured in Dulbecco's modified Eagle's medium (DMEM) supplemented with 10% fetal bovine serum (growth media). ATIC KO HeLa cells were cultured in DMEM supplemented with 10% FBS, 1% (v/v) penicillin/streptomycin, and 0.03 mm adenine. All cell lines were cultured in a humidified atmosphere of 5% CO_2_ at 37 °C. Experiments in hypoxia were carried out in a H35 Hypoxystation (Don Whitley Scientific) in which cells were cultured in humidified atmosphere of 1% O_2_, 5% CO_2_, and 94% nitrogen at 37 °C. For studies in purine-depleted conditions, HeLa cells were cultured in Roswell Park Memorial Institute (RPMI) medium supplemented with 5% of dialyzed fetal bovine serum for 1 week prior to initiation of experiments.

### Sample preparation for metabolomics analysis in cells cultured with isotope: [^13^C_6_]glucose

Growth media for isotope incorporation was prepared as follows on the day of the experiment: glucose-free glutamine free DMEM (A14430, Life Technologies, UK) was supplemented with 25 mm [^13^C_6_]glucose (CLM-1396-1, CK isotopes), 1 mm glutamine (GlutaMAX, 35050061, Life Technologies UK), and 10% FBS.

8 × 10^5^ HeLa cells maintained in DMEM + 10% FBS were plated in 100-mm dishes and incubated in normoxia for 24 h. The next day, media was removed, cells were washed once with PBS, and fresh warm media containing 25 mm [^13^C_6_]glucose was added to the dishes. Cells were subsequently placed in normoxia or hypoxia for 2 and 24 h. Following incubation media from these dishes was discarded, cells were washed twice with PBS, and 3 ml of warm trypsin-EDTA was added to the dishes to detach the cells. Once cells were detached, 7 ml of growth media was added to each dish, cell suspensions were collected into 15-ml conical tubes. Cell suspensions were then centrifuged at 160 × *g* for 4 min and pellets were washed three times with PBS. Supernatants were discarded and cell pellets were resuspended.

### Sample preparation for metabolomics analysis in cells cultured with isotope: [^15^N]glutamine

Growth media for isotope incorporation was prepared as follows on the day of experiment: glutamine-free DMEM (11960-044, Life Technologies, UK) was supplemented with 1 mm ^15^N-amide–labeled l-glutamine (NLM-557, CK isotopes) and 10% FBS (in purine-rich) or 10% dialyzed FBS (purine-depleted).

8 × 10^5^ HeLa cells maintained in DMEM + 10% FBS were plated in 100-mm dishes and incubated in normoxia for 24 h. The next day, media was removed, cells were washed once with PBS, and fresh warm media was added to the dishes. Cells were subsequently placed in normoxia or hypoxia for 12 h. Following incubation, 5 ml of media from one dish of each condition (purine-rich normoxia, purine-rich hypoxia, and purine-depleted normoxia) was transferred to a 15-ml centrifuge tube and kept aside for later treatment. The remaining media from these dishes was discarded, cells were washed twice with PBS, and 3 ml of warm trypsin-EDTA was added to the dishes to detach the cells. Meanwhile the media of the remaining dishes was removed, the cells were washed with PBS once, and [^15^N]glutamine-supplemented media was added to the cells. Cells were then allowed to incubate for the appropriate amount of time. Following incubation, the media was removed, the cells washed with PBS and trypsinized as before and treated as follow: once the cells detached, 7 ml of growth media was added to each dish and the cell concentration was measured in the cell suspension using a Moxi Z Mini Cell Counter (Orflo). Cell suspensions were then centrifuged at 160 × *g* for 4 min and pellets were washed three times with PBS. Supernatants were discarded and cell pellets were resuspended in 200 μl of ice-cold 100% methanol.

### Metabolites extraction for LC–MS

Metabolites extraction protocol was based on a previously reported experiment with some modifications (5). Methanol-quenched cell pellets were vortexed, snap frozen, and thawed on ice for 10 min. This freeze-thaw cycle was repeated two more times. Final suspensions were centrifuged for 15 min at 3,000 × *g*, supernatants were collected. This extraction process was repeated for each sample using 200 μl of 80% methanol in water. Combined supernatants from the two extractions were centrifuged for 30 min at 10,000 × *g* and supernatants were dried and reconstituted in 30 μl of 3% (v/v) methanol in water with 1 μm chlorpropamide and 0.1% (v/v) formic acid and analyzed by LC–MS using a modified version of an ion pairing reversed phase negative ion electrospray ionization method (53). Samples (10 μl) were separated on a Phenomenex Synergi Hydro-RP C18 column (100 × 2.1 mm, 2.5 μm particle size) using a water-methanol gradient. The LC–MS platform consisted of a Dionex Ultimate 3000 quaternary HPLC pump, a Dionex 3000 column compartment, a Dionex 3000 autosampler, and an Exactive Plus Orbitrap Mass Spectrometer controlled by Xcalibur 2.2 software. The HPLC column was maintained at 30 °C and a flow rate of 200 μl/min. Solvent A was 3% (v/v) aqueous methanol with 10 mm tributylamine and 15 mm acetic acid; solvent B was methanol. The gradient was 0 min at 0% solvent B; 5 min at 20% solvent B; 7.5 min at 20% solvent B; 13 min at 55% solvent B; 15.5 min at 95% solvent B; 18.5 min at 95% solvent B; 19.0 min at 0% solvent B; 25 min at 0% solvent B. The Exactive Plus was operated in negative ion mode at maximum resolution (140,000) and scanned from 70 to 1000 *m*/*z*.

Peak areas for all metabolites were calculated using Xcalibur 2.2 software. All spectra were aligned to one another and metabolites were identified using an in-house metabolite reference library. Peak areas were corrected for injection differences using chlorpropamide (internal standard) and normalized to the number of cells or represented as percent of total isotopologue species.

### Transient transfection for cell imaging

Cells were plated in 35-mm Petri dishes (Corning) at a confluence of 1.5 × 10^5^ cells/plate/day prior to transfection. Cells were transiently transfected (with plasmid(s) and/or siRNA) and incubated for 24 h. For two plasmid transfection and plasmid/siRNA co-transfection, cells were transfected using Lipofectamine 2000 (Invitrogen) in Opti-MEM I reduced serum media (Invitrogen) following the manufacturer's protocol. All other transfections were carried out with 5 μl of Lipofectamine 2000 and 1 µg of each DNA plasmid used per reaction; siRNA was used at a final concentration of 5 nM.

After 5 h of incubation with the Lipofectamine-DNA or Lipofectamine-DNA-siRNA complex, cells were washed with PBS and media was replaced with growth media. Cells were subsequently incubated in normoxia or hypoxia for 24 h unless stipulated otherwise.

### Cell treatments with DFX, 2-DG, 6-AN, Cpd14, and hypoxanthine

Following transfection, cells were washed with PBS and fresh warm media was added to the plates prior to compound treatment. Cells treated with DFX (100 μm) were incubated for 24 h in normoxia. Cells treated with Cpd14 (250 μm) were incubated for 24 h in hypoxia. Cells treated with hypoxanthine (60 μm) were incubated for 24 h in hypoxia. Cells treated with 2-DG (5 mm) or 6-AN (1 mm) were incubated for 30 min in normoxia, followed by 6 h of incubation in hypoxia.

### Synchronization of HeLa cells in G_1_ phase

HeLa cells transfected with FGAMS-EGFP were synchronized in G_1_ phase using dibutyryl-cAMP (Bt_2_cAMP, Sigma-Aldrich), as previously described (22). Briefly, 0.5 mm Bt_2_cAMP was added to the cell medium following transfection and cells were allowed to incubate for an additional 18 h in normoxia and hypoxia prior to live imaging.

### Live cell imaging

Cells were washed twice with Hanks' balanced salt solution (ThermoFisher Scientific) prior to imaging. All live samples were imaged at room temperature (∼25 °C) in Hanks' balanced salt solution using a 40× objective using an Axio Vert A1 Inverted microscope (Carl Zeiss Microscopy) and a HXP-120V light source (Carl Zeiss Microscopy). A Fs46 set (excitation 500/20 nm; emission 535/30 nm), a Fs31 filter set (excitation 565/30 nm; emission 620/60 nm), and a Fs02 filter set (excitation 365 nm; emission 420 nm) were used to accomplish the detection of GFP, mCherry, and DAPI, respectively. Image acquisition was done using ZEN software (Zeiss).

### Live recording of fluorescence microscopy

HeLa cells were plated in a glass-bottom dish and transfected with FGAMS-mCherry 16 h prior to the experiment. On the day of the experiment, cell media was changed for phenol red-free DMEM supplemented with 10% FBS. The dish was observed on a Deltavision Elite live imaging system equipped with a 37 °C incubator. HeLa cells were observed using a mCherry filter set. A gas bottle containing a hypoxic gas mixture (1% oxygen, 5% CO_2_, 94% nitrogen) was equipped with a gas regulator, a gas flowmeter set up at 20 ml/min, and gas tubing to inject a hypoxic environment directly into the cell dish. Recording was carried out for 4 h and a picture of the cell interest was taken every 15 min.

### Cell fixing and confocal fluorescent microscopy

Cells were cultured on a glass coverslip 22 × 22-mm placed in a 35-mm dish. Cells grown on the coverslips were washed twice with PBS and then fixed for 20 min at room temperature using freshly made 4% (w/v) paraformaldehyde in PBS. Cells were washed twice with PBS and the coverslip was mounted on a glass slide using DAPI mounting medium.

Fixed cells were imaged with a Nikon Plan Apochromat 63× oil-immersion objective on a Leica SP8 Inverted scanning confocal microscope. GFP was imaged using the 488 nm wavelength and PMT green detection. mCherry was imaged using a 561-nm solid-state laser line and PMT red detection. Each acquisition was averaged at least four times to eliminate background signals. Z-stacking was performed in 15 steps through the cell volume. DAPI was imaged using a 405 nm laser.

### Analysis of colocalization between mitochondria and purinosomes using SRRF

SRRF imaging of mitochondria and purinosomes was performed using a Nikon Plan Apochromat 63× oil-immersion objective on a Leica SP8 Inverted scanning confocal microscope. HeLa cells grown on glass coverslips were transfected with plasmid encoding FGAMS-EGFP and subsequently incubated in hypoxia. Mitochondria was stained with MitoTracker Deep Red dye (M22426, Thermo Fisher Scientific) following the manufacturer's protocol and cells were subsequently fixed and coverslip mounted on glass slides. GFP was imaged using the 488-nm argon laser line and PMT detection 518-550 nm (green). MitoTracker Deep Red was imaged using the 633 nm argon laser line and HyD detection 645-700 nm. 100 frames were acquired over time in the shortest possible time and the resulting pictures were then processed using NanoJ SRRF plugin on ImageJ (FIJI) software (45).

### Proximity ligation assay

All PLA experiments were performed according to the manufacturer's instructions, in 8-well chamber slides (C1782, Nunc Lab-Tek, Sigma-Aldrich). 2 × 10^4^ HeLa cells were plated per well to a final volume of 300 μl of growth media. Slides were incubated in normoxia or hypoxia for 24 h. Following incubation, media was removed from the wells and cells were washed twice with 200 μl of PBS. Cells were then fixed with 100 μl of 4% paraformaldehyde in PBS, at room temperature for 10 min. Following fixation, cells were washed once with PBS. Cells were then permeabilized using 200 μl of 0.5% Triton X-100 in PBS, at room temperature for 10 min. Permeabilizing solution was then removed and cells were washed with PBS, followed by the addition of a drop of Duolink Blocking Solution per well. Slides were incubated at room temperature for 1 h. Blocking solution was removed and cells were incubated overnight at 4 °C with 40 μl final volume of primary antibodies (diluted according to manufacturer's instructions). A negative control with only one primary antibody and a technical control with no antibody were included on each slide. After overnight incubation, primary antibodies solutions were removed. Slides were washed in Wash Buffer A for 2× 5 min, PLA probes (anti-mouse MINUS and anti-rabbit PLUS) were diluted 1:5 in antibody diluent, added to each sample. After incubation at 37 °C for 1 h, the slides were washed in fresh Wash Buffer A for 2× 5 min. Ligation solution was made by diluting ligation stock 1:5 and ligase 1:40 (1 μl final per sample) in the same solution of sterile H_2_O. Ligation solution was added to the sample and incubated for 30 min at 37 °C. Slides were washed for 2× 2 min with Wash Buffer A. Amplification polymerase solution was prepared by diluting amplification stock, 1:5, and polymerase, 1:80 (0.5 μl final per sample), in sterile H_2_O. This solution was added to each sample and incubated at 37 °C for 100 min. Slides were washed with Wash Buffer B (2× 10 min) followed by 1 min in 0.01× Wash Buffer B. The slides were then left to dry at room temperature and once dried, were mounted with Duolink In Situ Mounting Medium with DAPI. The edges of the coverslip were sealed and slides were observed on an Inverted scanning confocal microscope (Leica SP8). DAPI was imaged using a 405 nm laser and Duolink red dye was imaged using a 561-nm solid-state laser line and HyD detection 580-670 nm (red). When PLA was used for quantification, the minimum threshold for considering a cell to be positive was set at 6 signals per cell, based on the number of purinosome per cell determined in Fig. 1*e*.

### RNA extraction and qPCR

4.0 × 10^5^ cells were plated in 60-mm dishes and treated as detailed above. Total mRNA was extracted from cells using Reliaprep RNA miniprep kit (Promega). Briefly, cells were harvested using trypsin-EDTA, 0.05%, and subsequently diluted with growth media. Cells were collected and the solution was centrifuged at 160 × *g* for 4 min. Supernatants were discarded and pellets were washed twice with PBS. Pellets were then lysed in 100 μl of BL + TG buffer. Lysates were treated following the manufacturer's protocol. RNA was quantified using Nanodrop ND-1000 spectrophotometer. 1 µg of RNA was used as template to synthesize the complementary cDNA using GoScript Reverse Transcription kit (Promega) on a ThermoCycler PCR machine (Bio-Rad). qPCR was performed using TaqMan Universal Master Mix (Thermo Fisher Scientific) and TaqMan gene expression probes (Thermo Fisher Scientific). Expression values for each gene were expressed as ΔΔ*C_t_*. The expression of each studied gene was normalized to the average of 18S and β-actin. All expression values were normalized to normoxic gene expression.

### Western blotting analysis

HeLa cells were lysed by scraping with RIPA buffer (50 mm Tris, pH 7.4, 150 μm NaCl, 1 mm EDTA, 1% (v/v) Triton X-100) supplemented with cOmplete™ protease inhibitor mixture (Roche). Cell lysates were sonicated 10× 30/30 s in an ice-water bath and then centrifuged at 17,000 × *g* for 20 min at 4 °C. Protein concentration in each supernatant was quantified by Bradford assay. Samples were loaded onto an SDS-PAGE gel (20 to 50 µg/lane) to separate the proteins, which were subsequently transferred to a nitrocellulose membrane (GE Healthcare). The membranes were incubated with primary antibodies overnight at 4 °C. Antibodies were diluted in PBS, 0.1% Tween 20 supplemented with 5% powdered milk. Prior to incubating with secondary antibodies, membranes were washed three times using PBS, 0.1% Tween 20 (washing buffer). Secondary HRP-conjugated anti-rabbit and anti-mouse antibodies were diluted in 5% milk in PBS, 0.1% Tween 20 at 1:20,000 and 1:50,000 dilutions, respectively. β–Actin was used as internal control; HRP-conjugated anti-β-actin antibody was diluted (1:100,000) in 5% milk in PBS, 0.1% Tween 20. Membranes were incubated with HRP-conjugated antibodies for 1 h at room temperature. Band visualization was carried out using Amersham Biosciences ECL reagent (GE Healthcare, RPN2235) and imaging was processed using a ChemiDoc Imaging system linked to Image Laboratory 4.0 software (Bio-Rad).

### Cell growth measurement

HeLa cells were plated into 24-well plates at 5 × 10^4^ cells/well. Cells were allowed to recover overnight. The next day, cell number at time 0 was quantified. Briefly, media was removed, cells were washed once with DPBS. 200 μl of trypsin was added to each well. Cells were placed back into incubator until fully detached. 300 μl of warm growth media was added to each well. The cell concentration of each cell suspension was measured using Moxi Z Mini Cell Counter (Orflo) following the manufacturer's instructions. The remaining cells were placed in normoxia and hypoxia for 24 h. Following incubation, the same trypsinization and counting process was repeated. The cell concentration at 24 h was normalized to the cell concentration measured at 0 h.

### Single-cell analysis

To determine the number of clusters per cell as well as the diameter of each cluster, HeLa cells were transfected either with FGAMS-mCherry or ADSL-EGFP. The acquired pictures were analyzed using the “Analyze Particle” function in ImageJ as previously described (22).

### Data analysis

All statistical analysis was carried out on GraphPad Prism software using unpaired Student's *t* test. Statistical significance was defined as: *, *p* value < 0.05; **, *p* value < 0.01; ***, *p* value < 0.005; ****, *p* value < 0.0001. Z-stack and 3D volume reconstructions as well as Pearson's coefficient calculations were carried out using ImageJ software.

## Data availability

The data supporting the findings of this study are available within the paper and its supporting information files. Raw data underpinning this study are openly available from the University of Southampton repository (doi.org/10.5258/SOTON/D1384).

## Supplementary Material

Supporting Information
