# Supplementary material for: Hypoxia drives the assembly of the multienzyme purinosome complex
Source: J Biol Chem. 2020 May 21;295(28):9551–66. doi: 10.1074/jbc.RA119.012175 (PMC7363121; doi:10.1074/jbc.RA119.012175)
Supplement: Supporting Information [file supp_295_28_9551__index.html]

Hypoxia Drives the Assembly of the Multi-Enzyme Purinosome Complex — Purinosome Complex in Hypoxia — Hypoxia drives the assembly of the multienzyme purinosome complex — Purinosome complex in hypoxia — Supporting Information 

# Hypoxia drives the assembly of the multienzyme purinosome complex

## Supporting Information

- Supplementary Video 1 - Live formation of purinosomes in hypoxic HeLa cells observed using FGAMS- mCherry.
- Supplementary Figures - Supplementary Figures 1 to 5
