# Supplementary material for: Hypoxia drives the assembly of the multienzyme purinosome complex
Source: J Biol Chem. 2020 May 21;295(28):9551–66. doi: 10.1074/jbc.RA119.012175 (PMC7363121; doi:10.1074/jbc.RA119.012175)
Supplement: Supporting Information [file supp_RA119.012175_157500_3_supp_534559_q11sjr.pdf]

Supplementary Data

Hypoxia Drives the Assembly of the Multi-Enzyme Purinosome Complex

Cyrielle Doigneaux[1†], Anthony M. Pedley[2†], Ishna N. Mistry[1], Monika Papayova,[1] Stephen J. Benkovic[2], and Ali Tavassoli [1]*

From the [1]School of Chemistry, University of Southampton, Southampton, SO17 1BJ, U.K.; [2]Department of Chemistry, The Pennsylvania State University, University Park, PA 16802, U.S.A.

Running title: Purinosome Complex in Hypoxia

† These authors contributed equally.
*To whom correspondence should be addressed: Ali Tavassoli: School of Chemistry, University of Southampton, Southampton, SO17 1BJ, U.K. e-mail: a.tavassoli@soton.ac.uk

Keywords*:* Purinosome, Hypoxia, HIF-1, Metabolon, *de novo* purine biosynthesis.

Supplementary Figures

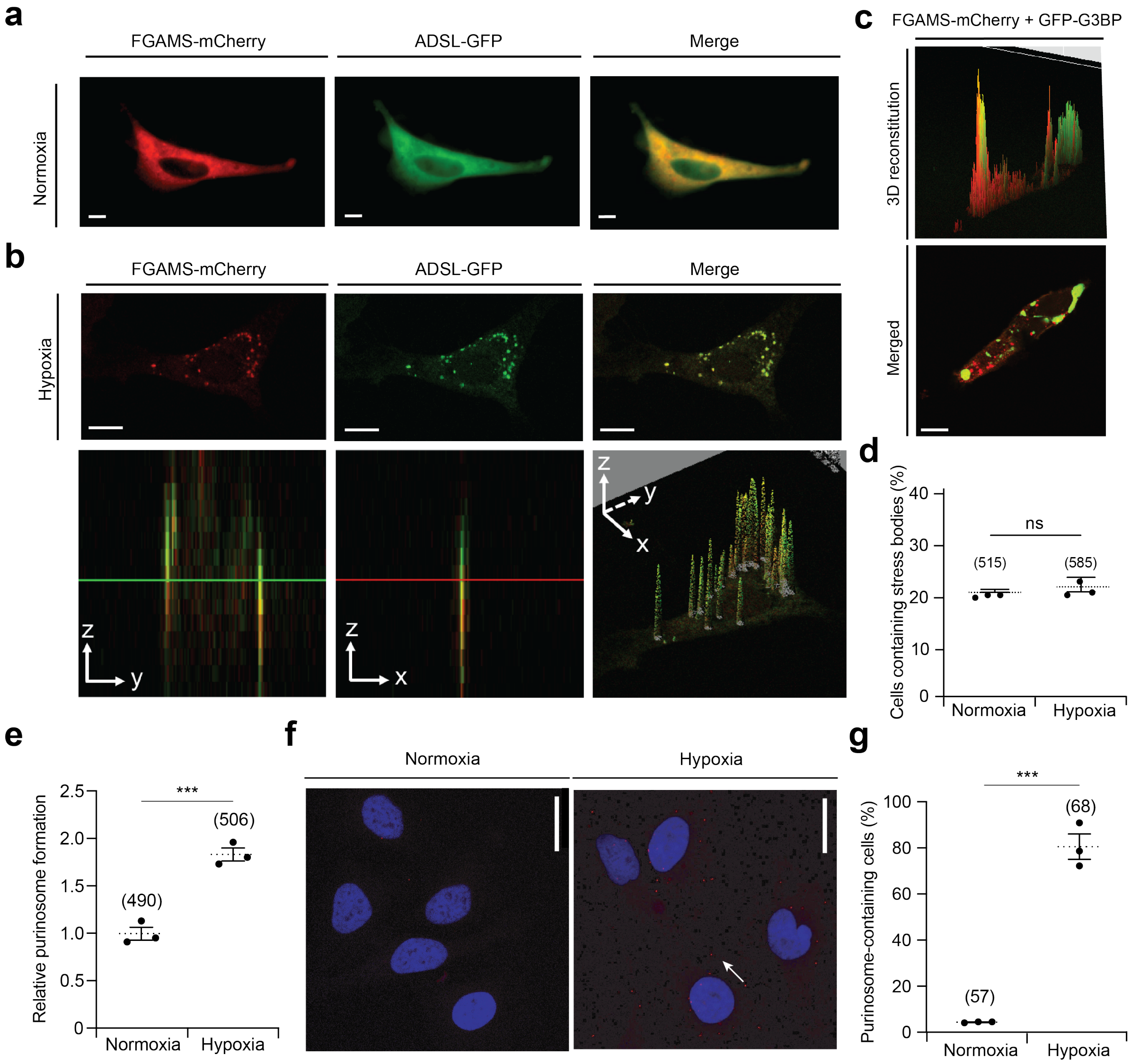

Figure S1. Assessing purinosome assembly in hypoxia. a) The clusters formed by FGAMS-mCherry overlaps with those formed by ADSL-EGFP in hypoxia. Even cytoplasmic distribution of the fluorescence signal for each enzyme in normoxia is also shown. Scale bar = 10 µm. b) Confocal microscope images showing the overlap of FGAMS-mCherry and ADSL-EGFP are shown along the z-y axis, z-x axis, and in the x-y-z view. Scale bar = 10 µm. c) The enzyme clusters formed by FGAMS-mCherry in hypoxic cells do not overlap with the stress-bodies formed by GFP-tagged G3BP. Scale bar = 10 µm. d) Hypoxia does not induce an increase in the number of cell-containing stress bodies. Data shown is n=3, ± S.E.M., total number of cells counted shown in parentheses. e) Quantifying purinosome formation in HeLa cells synchronised in G1 phase using FGAMS-EGFP in normoxia and hypoxia (18 h). Data shown is n=3, ± S.E.M., total number of cells counted shown in parentheses. f) The association of endogenous FGAMS and GART in hypoxia as measured by PLA (red spots; exemplar shown with a white arrow), with DAPI-stained nuclei in blue. Normoxic cells showed no PLA signal, PLA signal is observed in cells incubated in hypoxia. Scale bar = 25 µm, uncropped images are deposited in the raw data files. g) Quantification of cells showing a positive PLA signal in normoxia and hypoxia. Data shown are n=3, ± S.E.M., total number of cells counted shown in parentheses.

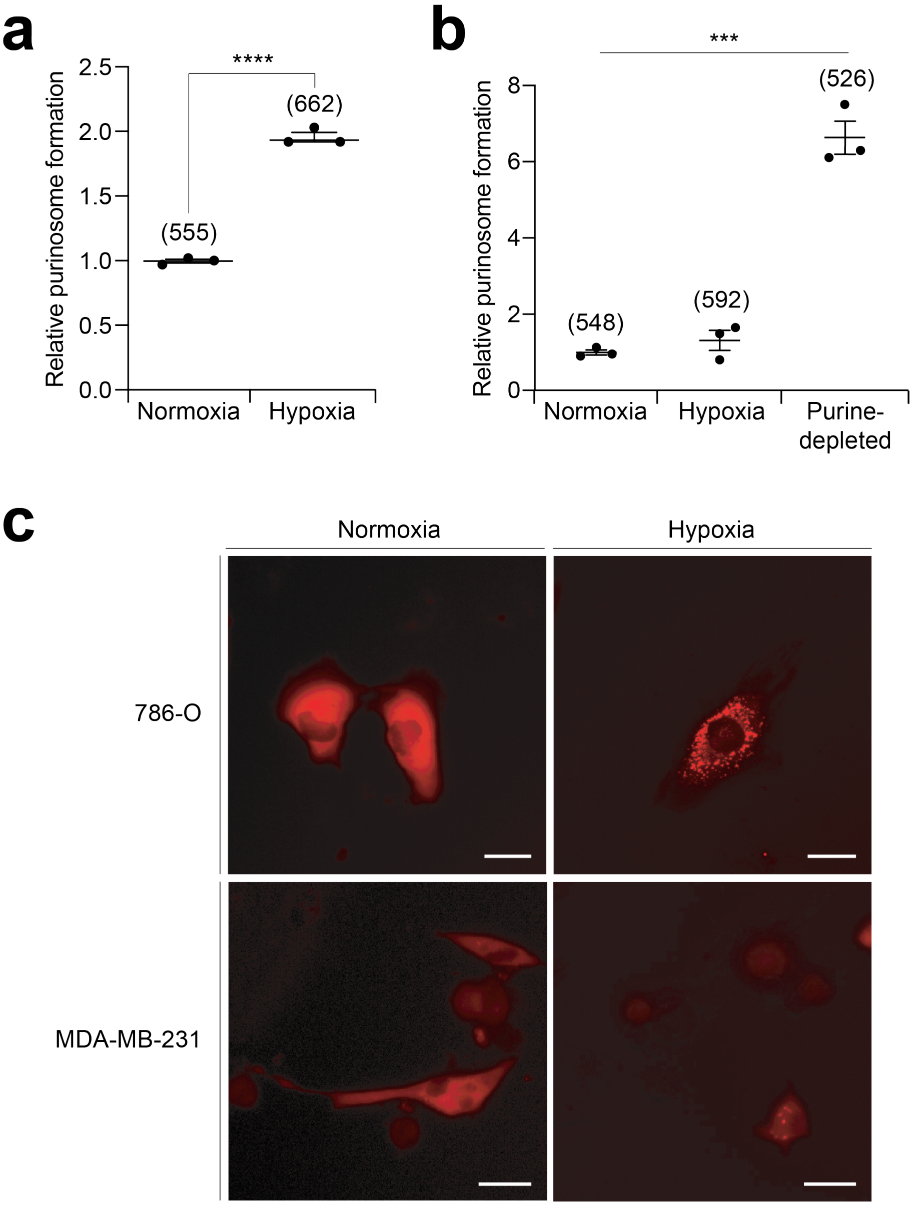

Figure S2. Purinosome formation in MDA-MB-231 and 786-O. a) Quantifying purinosome formation in MDA-MB-231 cells transfected with FGAMS-mCherry in normoxia and hypoxia (24 h). b) Quantifying purinosome formation in 786-O cells transfected with FGAMS-mCherry in normoxia purine-rich and purine-depleted as well as and hypoxia (24 h). Data shown is n=3, ± S.E.M., total number of cells counted shown in parentheses. c) Visualising purinosome formation using FGAMS-mCherry in 786-O and MDA-MB-231 cells. Fluorescent clusters can be observed in hypoxic cells. Scale bar = 25 μm.

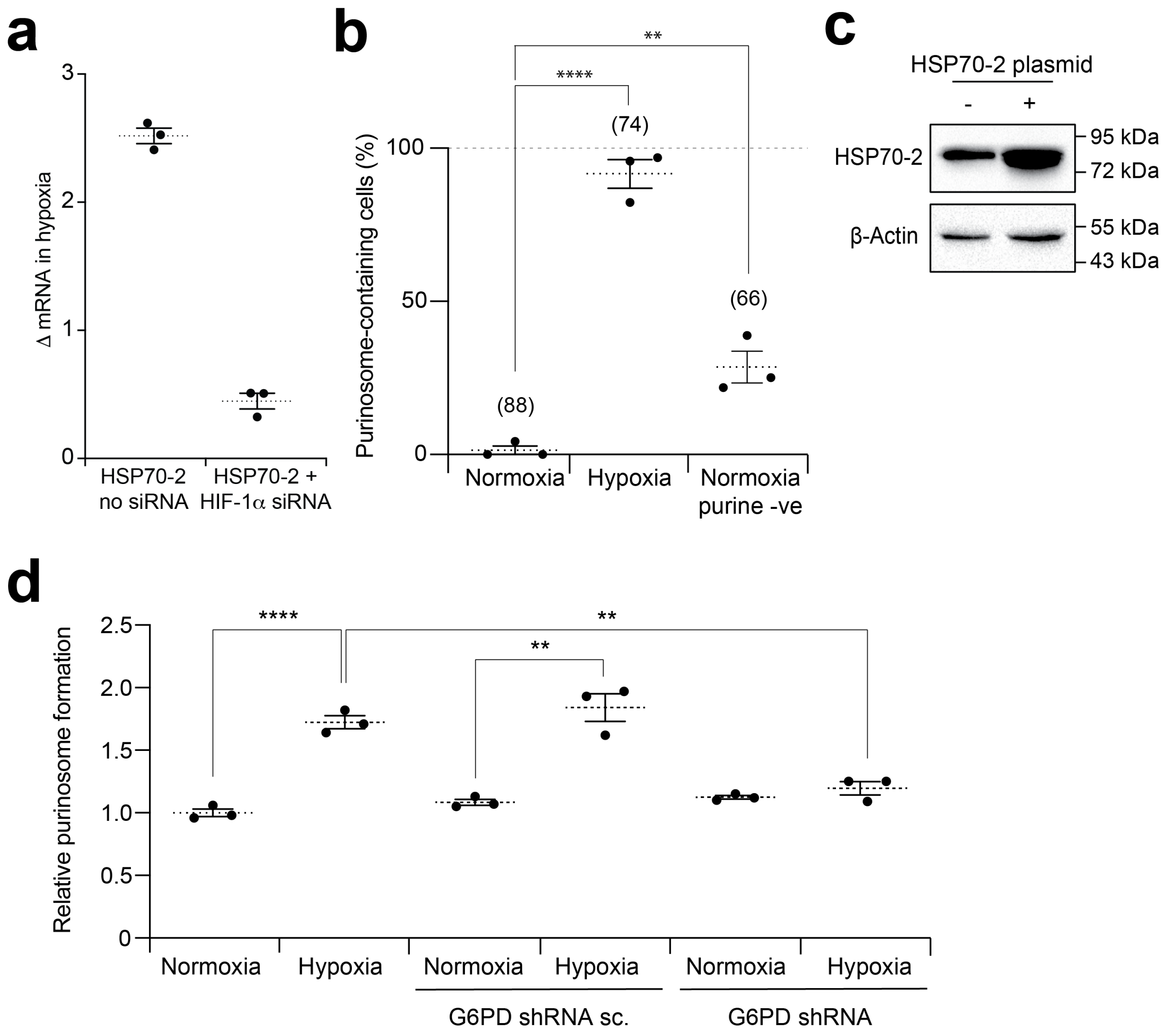

Figure S3. Effect of Hsp70-2 and G6PD-shRNA on purinosome formation. a) The effect of hypoxia (24 h) on the expression of *HSP70-2* in HeLa cells treated with HIF-1α siRNA measured by qPCR. Data shown is n=3, ± S.E.M. b) Quantifying the association of ADSL and HSP70-2 in normoxia and hypoxia in purine-rich medium and in normoxia in purine-depleted medium (purine -ve) by PLA. Data shown is n=3, ± S.E.M., total number of cells counted shown in parentheses. c) Overexpression of *HSP70-2* in HeLa cells from a plasmid leads to increased HSP70-2 protein levels. d) Quantifying purinosome formation in hypoxic HeLa cells (24 h) transfected with FGAMS-mCherry and treated with G6PD-shRNA or scrambled G6PD-shRNA (G6PD-shRNA sc.). The scrambled shRNA did not affect purinosome formation in hypoxia as a 1.8-fold was observed compared to normoxia and was similar to untreated cells. HeLa cells treated with G6PD-shRNA did not display an increased purinosome formation in hypoxia. Data shown is n=3, ± S.E.M.

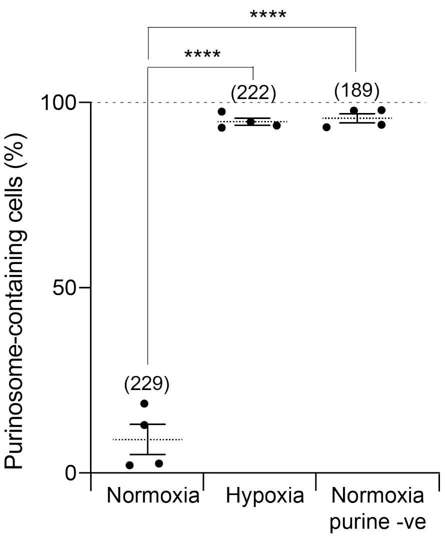

Figure S4. Quantifying the colocalisation between FGAMS and TOM20 by PLA. Quantification of the association of FGAMS and TOM20 in normoxia and hypoxia in purine-rich medium and in normoxia in purine-depleted medium (purine -ve) by PLA. Data shown is n=4, ± S.E.M., total number of cells counted shown in parentheses.

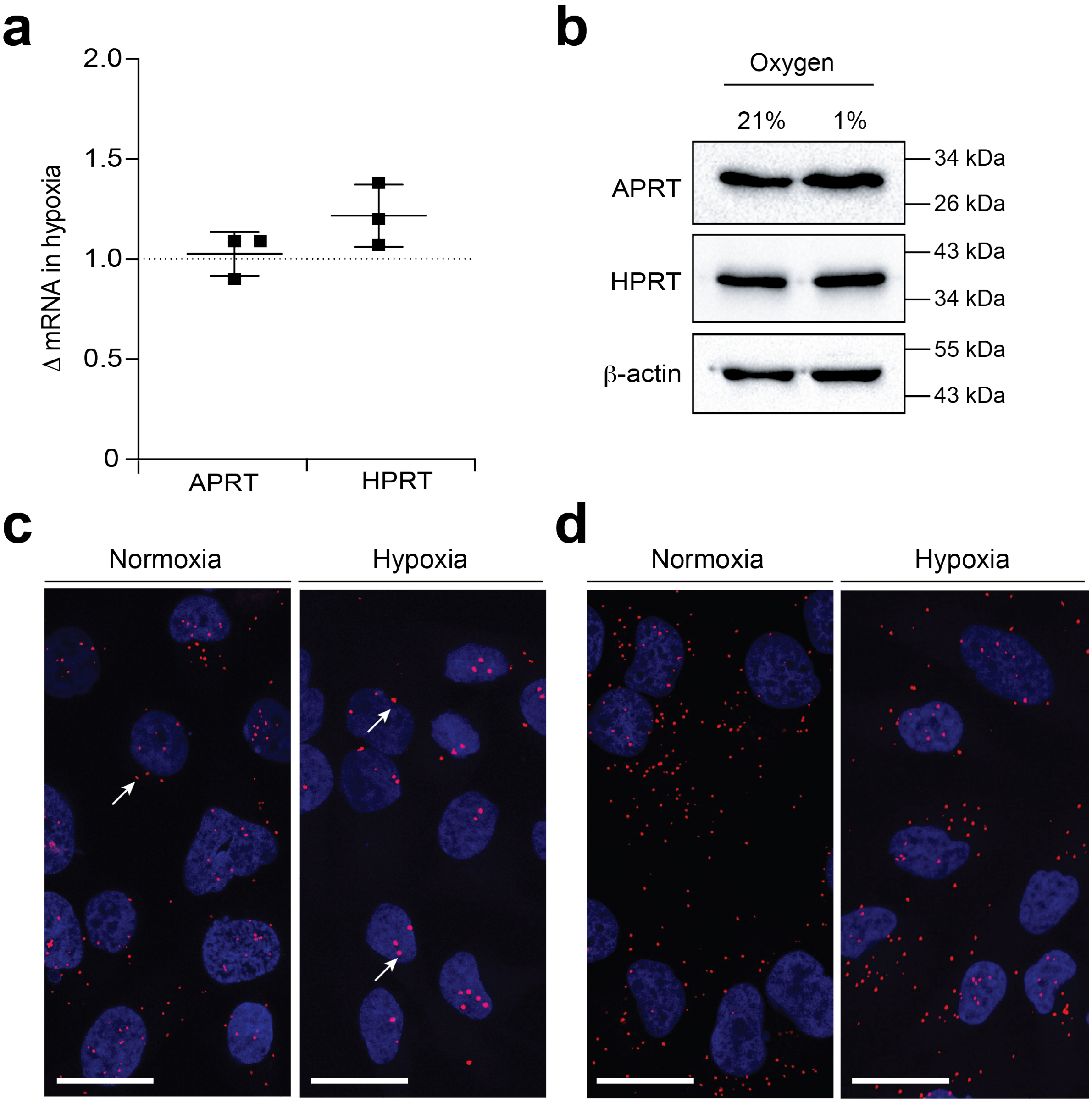

Fig. S5: Salvage enzymes are not affected in hypoxia. a) The effect of hypoxia on gene expression of the purine salvage pathway was measured by qPCR. The gene expression of APRT and HPRT remained unaffected in hypoxic conditions. Data shown is n=3, ± S.E.M. b) The effect of hypoxia on the protein levels of purine salvage enzymes. c) The association of endogenous ADSL and APRT in hypoxia as measured by PLA (red spots; exemplar shown with a white arrow), with DAPI-stained nuclei in blue. PLA signal is observed in cells incubated in both normoxia and hypoxia. d) The association of endogenous ADSL and HPRT in hypoxia as measured by PLA with DAPI-stained nuclei in blue. PLA signal is observed in cells incubated in both normoxia and hypoxia. Scale bar = 25 µm, uncropped images are deposited in the raw data files.

Supplementary Video

Video S1: Live formation of purinosomes in hypoxic HeLa cells observed using FGAMS-mCherry. HeLa cells transfected with FGAMS displayed homogenous fluorescence at the beginning of the live recording (t=0.15). Recording of the cellular localisation of FGAMS over time enabled to observe the formation of purinosomes within the cell cytoplasm from approximately 2 hours and 30 minutes. Purinosomes complexes remained formed until the end of the recording.
